# Fluorescent Crimean-Congo hemorrhagic fever virus illuminates tissue tropism patterns and identifies early mononuclear phagocytic cell targets in Ifnar^-/-^ mice

**DOI:** 10.1371/journal.ppat.1008183

**Published:** 2019-12-02

**Authors:** Stephen R. Welch, Jana M. Ritter, Anita K. McElroy, Jessica R. Harmon, JoAnn D. Coleman-McCray, Florine E. M. Scholte, Gary P. Kobinger, Éric Bergeron, Sherif R. Zaki, Stuart T. Nichol, Jessica R. Spengler, Christina F. Spiropoulou

**Affiliations:** 1 Viral Special Pathogens Branch, Division of High-Consequence Pathogens and Pathology, Centers for Disease Control and Prevention, Atlanta, Georgia, United States of America; 2 Infectious Diseases Pathology Branch, Division of High-Consequence Pathogens and Pathology, Centers for Disease Control and Prevention, Atlanta, Georgia, United States of America; 3 Division of Pediatric Infectious Disease, University of Pittsburgh School of Medicine, UPMC Children’s Hospital of Pittsburgh, Center for Vaccine Research Pittsburgh, Pennsylvania, United States of America; 4 Department of Microbiology, Immunology and Infectious Diseases, Université Laval, Quebec City, Quebec, Canada; Federal Ministry of Education and Research, GERMANY

## Abstract

Crimean-Congo hemorrhagic fever virus (CCHFV, order *Bunyavirales*, family *Nairoviridae*, genus *Orthonairovirus*) is the tick-borne etiological agent of Crimean-Congo hemorrhagic fever (CCHF) in humans. Animals are generally susceptible to CCHFV infection but refractory to disease. Small animal models are limited to interferon-deficient mice, that develop acute fatal disease following infection. Here, using a ZsGreen1- (ZsG) expressing reporter virus (CCHFV/ZsG), we examine tissue tropism and dissemination of virus in interferon-α/β receptor knock-out (Ifnar^-/-^) mice. We demonstrate that CCHFV/ZsG retains in vivo pathogenicity comparable to wild-type virus. Interestingly, despite high levels of viral RNA in all organs assessed, 2 distribution patterns of infection were observed by both fluorescence and immunohistochemistry (IHC), corresponding to the permissiveness of organ tissues. To further investigate viral dissemination and to temporally define cellular targets of CCHFV in vivo, mice were serially euthanized at different stages of disease. Flow cytometry was used to characterize CCHFV-associated alterations in hematopoietic cell populations and to classify infected cells in the blood, lymph node, spleen, and liver. ZsG signal indicated that mononuclear phagocytic cells in the lymphatic tissues were early targets of infection; in late-stage infection, overall, the highest levels of signal were detected in the liver, and ZsG was found in both antigen-presenting and lymphocyte cell populations.

## Author summary

Human infection by tick-borne Crimean-Congo hemorrhagic fever virus (CCHFV) can result in severe disease with up to 30% case fatality rates. While CCHFV is known to be hepatotropic, the presence and implications of virus in other tissues are less clear. Furthermore, to date, early cellular targets of infection in a CCHFV disease model have not been investigated in detail. Here, using a recombinant reporter CCHFV expressing the fluorescent protein ZsGreen1 (ZsG; CCHFV/ZsG) in interferon-α/β receptor knock-out (Ifnar^-/-^) mice, which develop acute fatal disease following infection, we investigate both cellular and tissue targets of infection. Importantly, we find that CCHFV/ZsG infection demonstrated comparable pathogenicity to wild-type virus in Ifnar^-/-^ mice. We used in situ visualization of fluorescent signal in tissues to assess viral dissemination throughout the course of infection, and found robust viral signal in reproductive tissues, previously unrecognized as sites of CCHFV infection. We also used flow cytometry to detect intracellular fluorescent signal, and identified initial target cells of CCHFV infection as macrophage and monocyte populations in lymphatic tissues. These findings support a central role of immune cells in early virus dissemination, and a need for further investigations into reproductive tract involvement in human CCHFV infection.

## Introduction

Crimean-Congo hemorrhagic fever virus (CCHFV, order *Bunyavirales*, family *Nairoviridae*, genus *Orthonairovirus*) is the tick-borne etiological agent of Crimean-Congo hemorrhagic fever (CCHF) in humans. Endemic in regions of Europe, Africa, the Middle East, and Asia, the virus is maintained in nature via a tick-vertebrate-tick cycle of transmission involving both small mammals and large ungulates [1–3]. Human infections occur via tick bites, nosocomial transmission, or direct contact with infected tissues, the latter generally through abattoir or veterinary procedures. Human disease typically presents as a non-specific febrile illness, but can progress to hemorrhagic manifestations in severe cases, with case fatality rates ranging 5–30%.

As a high-consequence pathogen with an expanding geographic range, CCHFV is increasingly recognized as a growing public health concern [4]. The availability of disease models remains a challenge in CCHFV research; lethal models are currently limited to interferon-(IFN) deficient (Stat-1^-/-^ and Ifnar^-/-^) mice [5–7], and humanized (Hu-NSG-SGM3) [8] mice, although disease has been recently described in cynomolgus macaques [9]. Despite their limitations, IFN-deficient mice have been instrumental in facilitating the study of CCHFV pathogenesis in vivo. To date, dissemination of viral RNA and antigen in susceptible animals has been described using traditional techniques, such as PCR, immunohistochemistry (IHC), and in situ hybridization [7,8,10]. A limitation to studies using antibody-based staining techniques is the need for efficient co-staining processes to recognize and differentiate CCHFV epitopes from cellular epitopes; such studies can be challenging to both perform and interpret. Recently, we engineered CCHFV/ZsG, a reporter CCHFV expressing the fluorescent protein ZsGreen1 (ZsG) [11]. Cells infected with CCHFV/ZsG express quantitative levels of ZsG, with expression solely dependent on authentic viral replicational and transcriptional processes. Direct detection and visualization of infected cells by fluorescent protein expression significantly reduces the technical aspects of both IHC and flow cytometry. The advantages to this approach have been highlighted in key studies using fluorescent recombinant reporter viruses in animal models of disease to investigate cellular targets of infection, tissue tropism, and viral dissemination for multiple pathogens including measles [12], henipaviruses [13], and influenza viruses [14].

In this study, we confirmed that infection with reporter CCHFV/ZsG is consistent with wild-type virus infection in the established Ifnar^-/-^ mouse model [7]. Gross in situ imaging data support the hepatotropic nature of CCHFV, with robust viral replication in the liver in end-stage disease. However, other tissues not previously recognized as CCHFV targets, including the female reproductive tract, were also noted to have high levels of infection. Furthermore, using flow cytometry, monocyte/macrophage cells in the draining lymph node were identified as the first detectably infected cell populations. CCHFV was also found in monocyte/macrophage cells later in infection, as well as in other antigen-presenting cell populations in spleen and in the liver.

## Results

### Pathogenicity of ZsG-expressing reporter virus in Ifnar^-/-^ mice is similar to that of wild-type CCHFV

**Fig 1:**
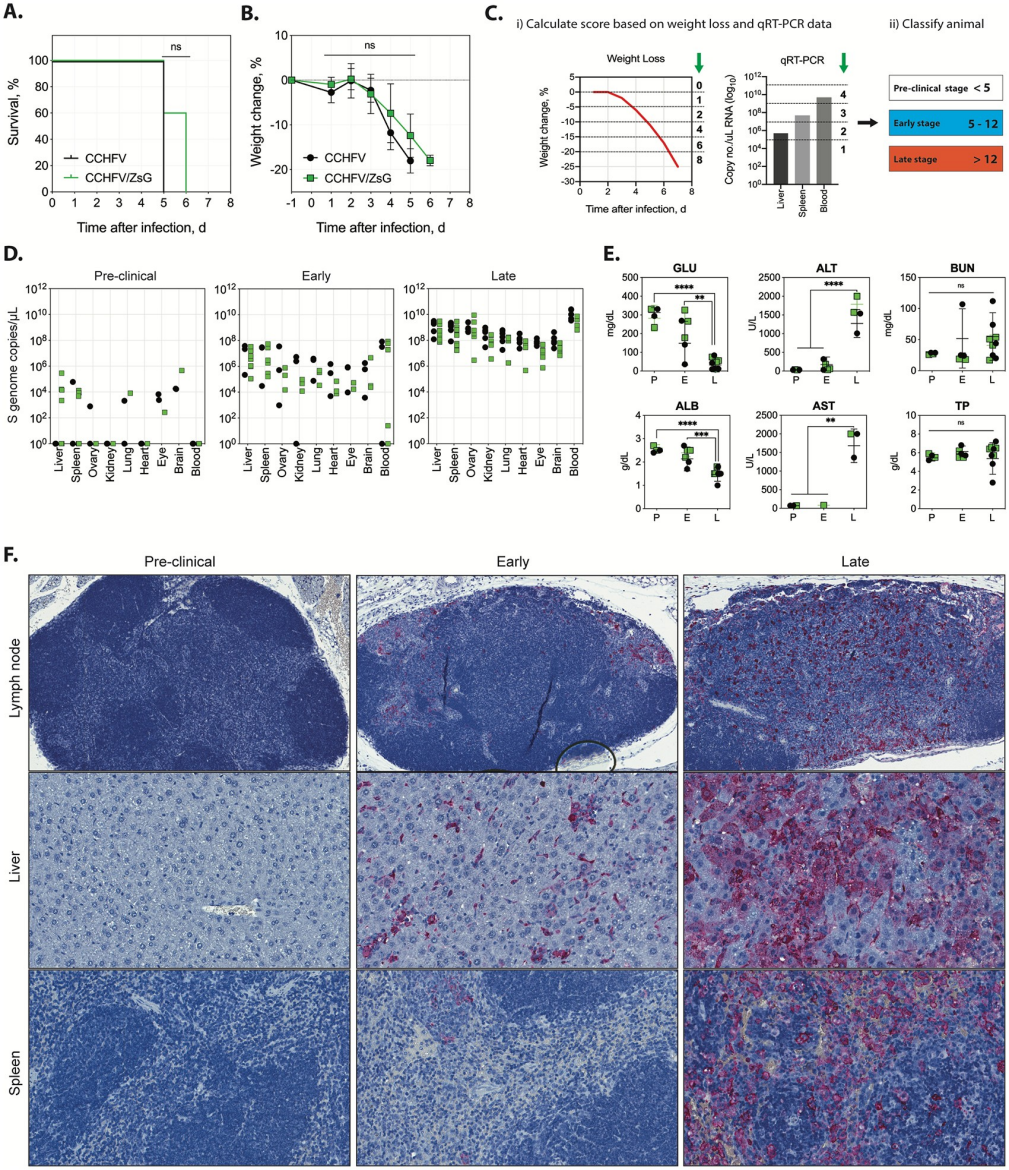
Comparative infections of wild-type CCHFV and reporter CCHFV/ZsG. (A) Survival and (B) weight change in Ifnar^-/-^ mice inoculated subcutaneously with 100 TCID_50_ recombinant wild-type CCHFV (CCHFV; black line with circles; *n* = 5) or recombinant CCHFV expressing ZsG (CCHFV/ZsG; green line with squares; *n* = 5). Lines represent mean weight change of all individuals on that day; error bars represent ±SD. ns = not significant. (C) Mice were classified into one of 3 disease stage groups based on weight loss and viral RNA levels in liver, spleen, and blood determined by qRT-PCR. Weight loss scoring criteria: 0 to -5% = 1; -6 to -10% = 2; -11 to 15% = 4; -16 to 20% = 6; > -20% = 8. Viral load scoring criteria (values are CCHFV S segment copies/μL): <1 × 10^5^ = 1; <1 × 10^7^ = 2; <1 × 10^9^ = 3; <1 × 10^11^ = 4. Classification scores: pre-clinical stage: <5; early-stage disease: 5 to 12; late-stage disease: >12. (D) Quantitative RT-PCR was performed based on primer and probe sets specific for CCHFV nucleoprotein (NP) on tissues taken from animals infected with CCHFV (black circles) or CCHFV/ZsG (green squares). Disease severity in animals was classified as either pre-clinical, early, or late using a scoring system based on weight loss and viral tissue load (S1 Table). (E) Clinical chemistry values performed on whole blood samples collected at the time of euthanasia in CCHFV- (black circles) or CCHFV/ZsG- (green squares) infected mice classified as being in pre-clinical (P), early (E), or late (L) stage of disease. Individual values are represented, with means and standard deviation shown. GLU, glucose; ALB, albumin; ALT, alanine aminotransferase; AST, aspartate aminotransferase; BUN, blood urea nitrogen; TP, total protein. ns = not significant; * *p < 0*.*05*, ** *p < 0*.*01*, *** *p < 0*.*001*, **** *p < 0*.*0001*. (F) CCHFV NP immunostaining demonstrated a progressive increase in antigen expression at pre-clinical, early-, and late-stage disease in draining (axillary) lymph node, liver, and spleen of representative CCHFV/ZsG-infected Ifnar^-/-^ mice. Survival statistics were calculated using the Mantel-Cox test, weight statistics were calculated using multiple t-tests with Holm-Siddack’s multiple comparison test, and clinical chemistry statistics were calculated using two-way ANOVA with Tukey’s multiple comparison test.

Reporter viruses are often attenuated in vivo compared to the parental wild-type strain. Thus, to compare the in vivo pathogenicity of CCHFV/ZsG to that of the parental virus, Ifnar^-/-^ mice were inoculated subcutaneously (SC) with 100 tissue culture infective dose 50 (TCID_50_) of either wild-type CCHFV (*n* = 5) or CCHFV/ZsG (*n* = 5). Similar to reports of wild-type infection in immunodeficient mice [6,7], CCHFV- and CCHFV/ZsG-infected mice reached end-point criteria 5–6 days post infection (dpi) (Fig 1A; mean time to death = 5.6 dpi), and demonstrated analogous clinical signs (i.e., weight loss [Fig 1B], hunched posture, ruffled fur, and decreased activity).

To further characterize disease progression, Ifnar^-/-^ mice were inoculated SC with 100 TCID_50_ of either wild-type CCHFV (*n* = 6) or CCHFV/ZsG (*n* = 18), and sacrificed at 2, 4, or 5 dpi. In this subsequent study, Ifnar^-/-^ mice infected with either virus reached end-stage disease 4 or 5 dpi. Despite the short clinical course, at each sampled time point a spectrum of disease was observed as reflected in clinical signs (e.g., weight loss), viral RNA load in blood and tissues, and gross ZsG expression in organs. Within this spectrum, we found that data grouped more consistently by objective clinical signs (weight loss) and viral detection post-mortem (levels of viral RNA in blood and tissues) than by time post infection. Thus, to more accurately indicate the state of disease at time of sampling, we developed a scoring system based on these data (Fig 1C), which supported delineation of the disease course in infected Ifnar^-/-^ mice into 3 phases of disease: pre-clinical, early-stage, and late-stage. Details of the scoring system and corresponding groups are found in S1 Table. Pre-clinical disease, seen in 8 mice (all euthanized at 2 dpi), was defined by minimal weight loss, absence of clinical signs, and low-level viral RNA detection in some tissues. Early-stage disease, seen in 10 mice (2 sacrificed at 2 dpi, 7 at 4 dpi, and 1 at 5 dpi), was defined by up to 10% weight loss from baseline, hypoactivity, rising viral RNA levels in organs (10–100 fold higher than pre-clinical), and moderate viral RNAemia (10^1^–10^7^ copies/μL). Late-stage disease, seen in 16 mice (3 sacrificed or found dead at 4 dpi, 10 at 5 dpi, and 3 at 6 dpi), was defined by >10% weight loss from baseline, extensive viral RNA detection in tissues and blood (>10^8^ copies/μL), and severe clinical signs or death.

**Fig 2:**
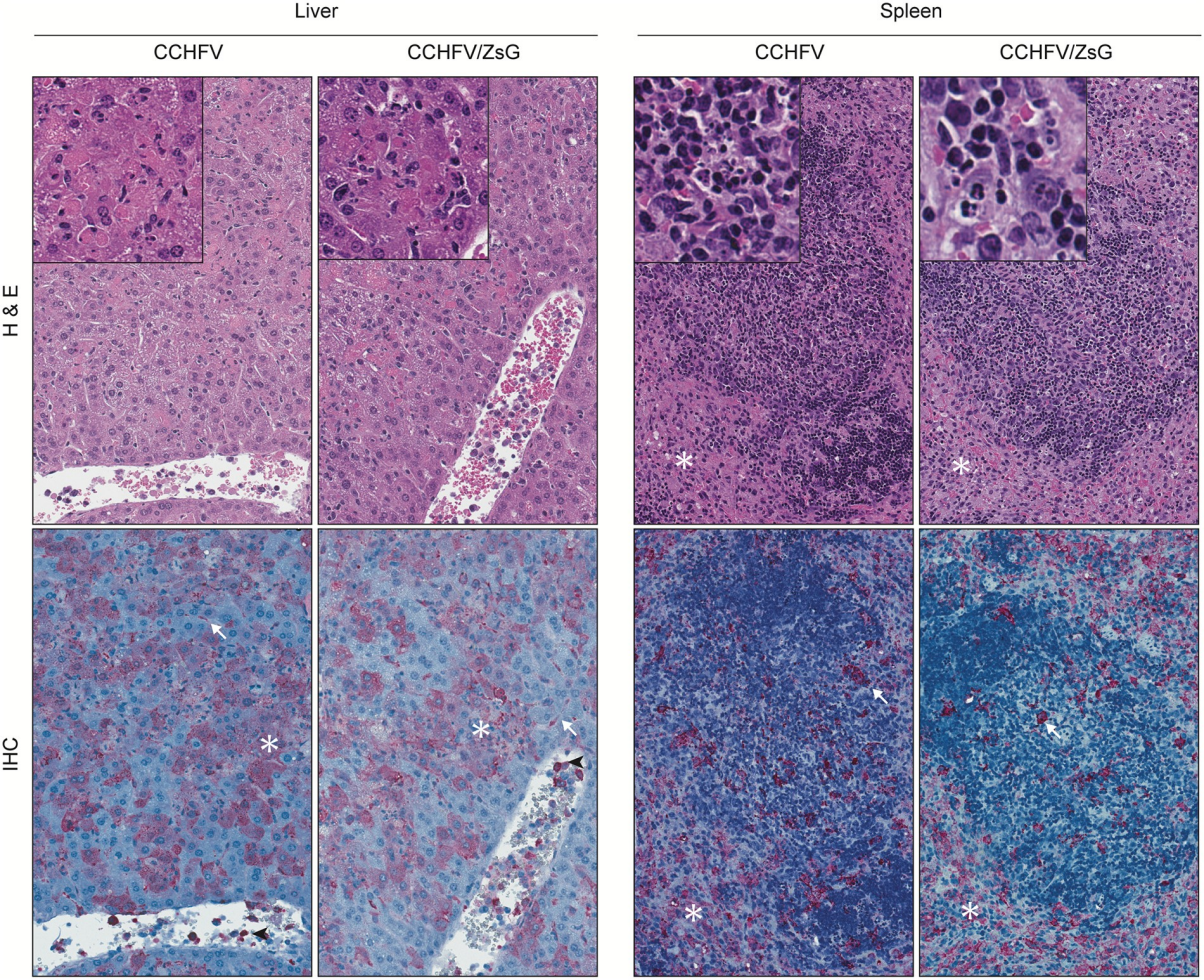
Liver and spleen pathology and CCHFV distribution visualized by immunohistochemistry in mice euthanized at 5 dpi. Mice displayed similarly severe pathology with abundant CCHFV immunostaining. Livers had widespread, random, and confluent hepatocellular necrosis (see insets) with minimal inflammation. Central veins of these mice contained increased numbers of leukocytes. CCHFV was detectable by immunostaining within necrotic and viable hepatocytes (✱), sinusoidal lining cells (arrows) including Kupffer cells and endothelium, and intravascular leukocytes (arrowheads). In spleens, lymphocytolysis (see insets) and red pulp expansion by histiocytes (✱) were observed, and immunostaining showed scattered CCHFV within lymphoid follicles (arrows) and extensive within red pulp histiocytes (✱). H&E, hematoxylin-eosin staining; IHC, CCHFV immunoalkaline phosphatase staining with naphthol fast red substrate, hematoxylin counterstain. Original magnification: liver, 200×; spleen, 200×.

Viral RNA levels in all tissues (Fig 1D) and clinical chemistry values (Fig 1E) were comparable between CCHFV- and CCHFV/ZsG-infected mice in the same disease stage. Decreases in both glucose (7.5-fold decrease) and albumin (1.7-fold decrease) were observed in animals in late-stage relative to early disease. Increases in mean alanine aminotransferase (ALT; 15-fold) and aspartate aminotransferase (AST; 22-fold) were seen in animals in late-stage relative to both pre-clinical and early disease. Other alterations included increased blood urea nitrogen (BUN) in some CCHFV- and CCHFV/ZsG infected mice in early- and late-stage disease. Disease progression was associated with increased antigen detection in organs of CCHFV/ZsG-infected mice (Fig 1F and S1 Fig). During the pre-clinical stage, CCHFV nucleoprotein (NP) antigen was not detected in any organs examined. In early-stage disease, antigen localization was multifocal and scattered, and in late-stage disease, antigen was more extensively distributed. Analogous antigen levels and distribution were observed in all organs examined from wild-type infected mice (S2 Fig). Furthermore, antigen distribution and histopathology in hepatic and splenic tissues from late-stage mice were comparable in mice infected with wild-type CCHFV and CCHFV/ZsG (Fig 2). Livers had hepatic necrosis with minimal inflammation and increased intravascular leukocytes. Antigen was detected in hepatocytes, sinusoidal lining cells including Kupffer cells and endothelium, and intravascular leukocytes. Spleens had lymphocytolysis and increased red pulp histiocytes, with antigen localization to macrophages.

### Widespread ZsG signal is detectible in situ, especially in liver, secondary lymphoid, and reproductive tissues

**Fig 3:**
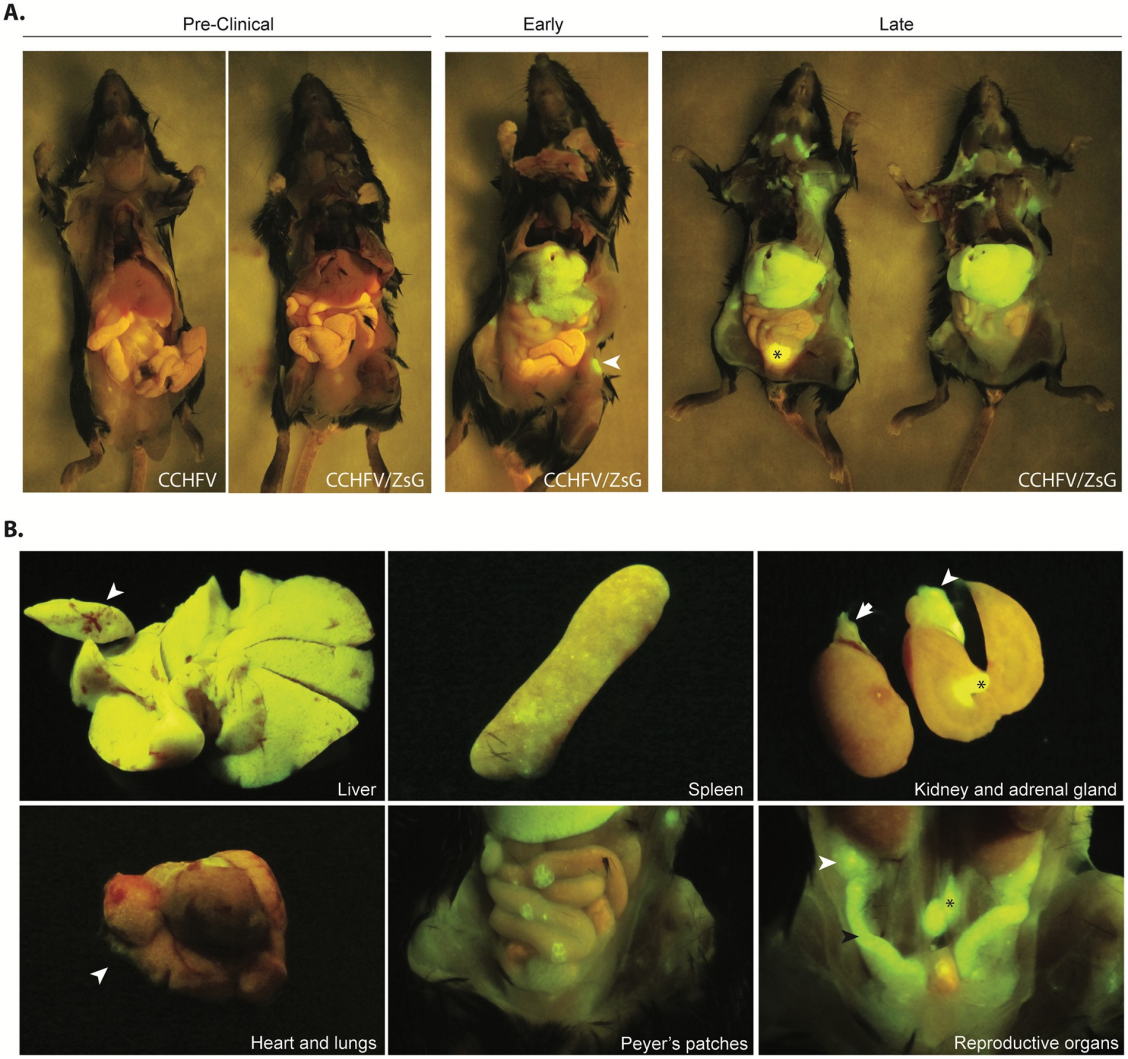
In situ visualization of CCHFV/ZsG infection. (A) No ZsG fluorescence was observed in pre-clinical CCHFV/ZsG-infected animals. A mouse infected with wild-type CCHFV is shown to demonstrate background autofluorescence levels when using the fluorescence detection setup; strong autofluorescence is seen in the bladder (due to urine content, see ✱ in right panel), and mild autofluorescence is observed in the intestinal tract. In early-stage CCHFV/ZsG infection, extensive ZsG fluorescence was detected in the livers and lymph nodes of the mice; white arrow head indicates inguinal lymph node. In late-stage CCHFV/ZsG infection, extensive ZsG fluorescence was observed in the liver and lymphoid organs (brachial, mediastinal, inguinal, and lumbar lymph nodes). A single representative animal from each clinical stage is shown; ZsG fluorescence levels in organs were similar between animals. (B) Organ-specific viral distribution patterns were observed in: liver (top left; white arrow represents a transverse cross section of one lobe of the liver); spleen (top middle); kidney and adrenal gland (top right, white arrow represents the adrenal gland, ✱ represents the renal pelvis demonstrating urine autofluorescence); heart and lung (bottom left; white arrow shows diffuse ZsG fluorescence in the lung); intestinal tract showing strong fluorescence in the Peyer’s patches (bottom middle); and reproductive tract (bottom right; white arrow, ovary; black arrow, uterus; ✱, iliac lymph nodes).

To investigate grossly the relative viral tissue distribution and patterns of infection, ZsG fluorescence was visualized in situ at time of euthanasia in CCHFV/ZsG-infected animals. Wild-type CCHFV-infected mice were used to assess levels of background autofluorescence, which was found to be distinguishable from ZsG fluorescence by its muted and markedly yellow presentation; autofluorescence was mild and generalized in the intestines, and moderate to high in the renal pelvis or bladder depending on urine content (Fig 3A, denoted by asterisk). In general, the presence and intensity of ZsG fluorescence was directly associated with NP antigen immunostaining (Fig 1F, S1 Fig) and viral RNA detection by qRT-PCR (Fig 1D). In pre-clinical mice, ZsG fluorescence was not detected in any tissues (Fig 3A, left panel). In early-stage disease, strong fluorescence was seen in both the liver and in multiple peripheral lymph nodes, including cervical and inguinal lymph nodes (Fig 3A, middle panel). In late-stage disease, fluorescent signal was noticeably stronger than at previous stages and widely disseminated in various organs, including lymph nodes, liver, spleen, kidneys, lung, adrenal gland, gastrointestinal tract, and reproductive organs (Fig 3A, right panel). Grossly, livers of both CCHFV- and CCHFV/ZsG-infected mice euthanized at both early- and late-stage were comparatively paler on examination than livers from the pre-clinical animals. Signal distribution patterns within organs varied. In late-stage, intense diffuse signal was observed in the liver, with cross-sections revealing that the vast majority of cells expressed ZsG (Fig 3B, top left panel). ZsG fluorescence was multifocal and intense in the spleen (Fig 3B, top middle panel) and adrenal gland (Fig 3B, top right panel), with only mild fluorescence observed in the kidney. Mild multifocal fluorescence was seen in the lungs and pericardium (Fig 3B, bottom left panel). Fluorescence in the gastrointestinal tract was focal in areas of lymphoid tissue (Peyer’s patches, Fig 3B, lower middle panel). Additionally, intense diffuse fluorescence was observed in the reproductive tissues (ovary and uterus, Fig 3B, lower right panel). With the exception of liver and lymphoid tissues, ZsG fluorescence correlated with NP immunostaining without overt pathology.

### Organ distribution and intensity of ZsG signal in situ are associated with 2 histological patterns of infection

**Fig 4:**
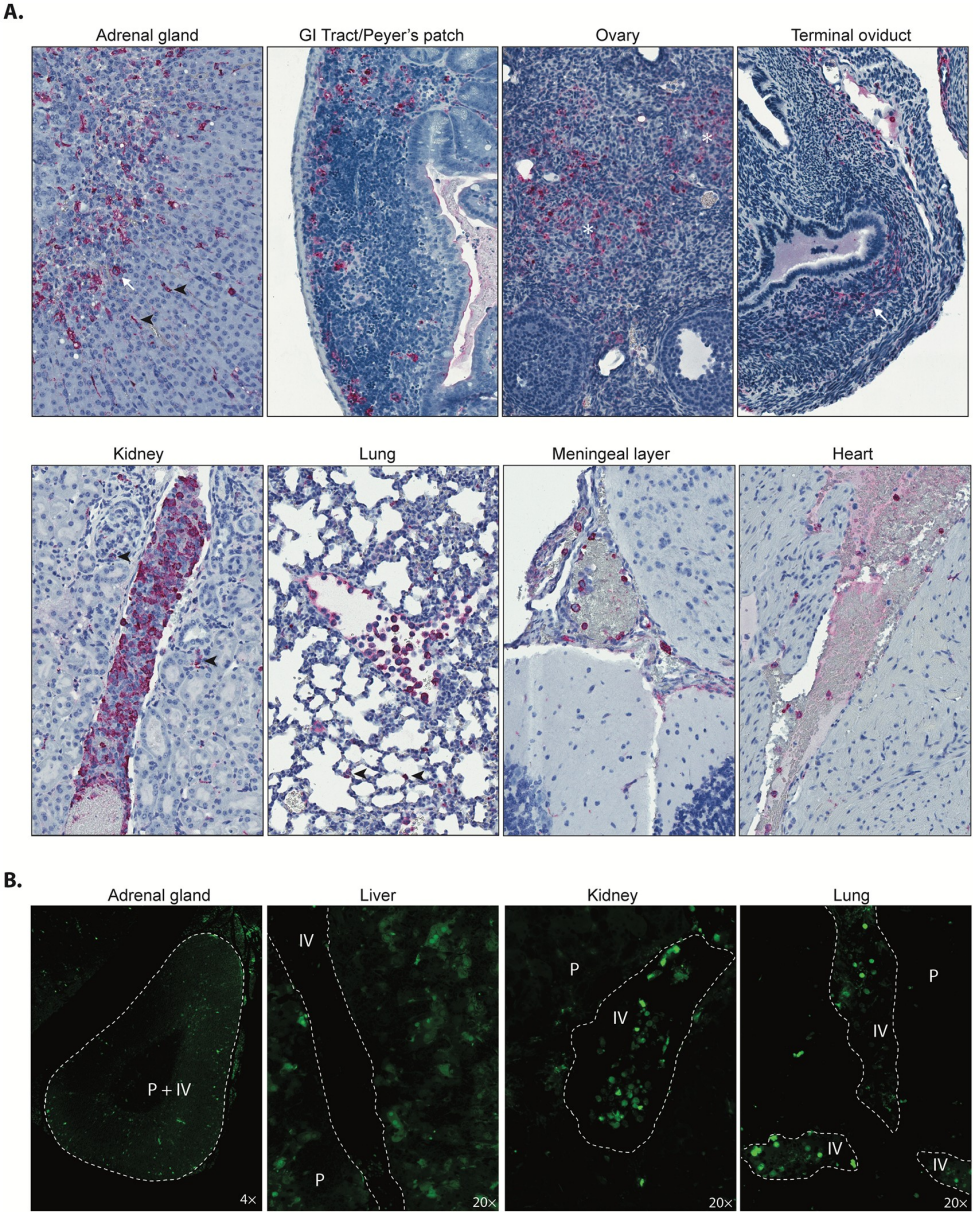
Distribution of viral NP antigen visualized by immunohistochemistry (IHC) in a CCHFV/ZsG-infected Ifnar^−/−^ mouse at 6 dpi. (A) NP immunostaining in parenchymal and lymphoid tissues (top row). NP was visualized in epithelial cells (arrow) and endothelial/intravascular cells (arrowheads), most prominently at the adrenal corticomedullary junction, and scattered staining was seen in macrophages in gut-associated lymphoid tissue (Peyer’s patches). Focal staining was also observed in the ovarian medullary stroma (✱) and in the lamina propria of the terminal oviduct (arrow). NP immunostaining within intravascular leukocytes in various organs (bottom row). Increased intravascular leukocytes with abundant immunostaining were seen in kidney and lung, with rare staining of intracapillary leukocytes and interstitial cells (arrowheads). In the brain and heart, NP was detected in leukocytes within leptomeningeal vessels and in the lumen of the right heart ventricle. IHC: NP immunoalkaline phosphatase staining with naphthol fast red substrate, hematoxylin counterstain. Original magnification: 200×. (B) ZsG fluorescence in embedded fixed issues. Parenchymal and intravascular ZsG signal for infected cells can be visualized without immunostaining using a fluorescent microscope: P = parenchyma (tissue); IV = intravascular; dotted line represents the boundary between the blood vessel and the surrounding tissue.

Despite comparable levels of virus detection by qRT-PCR in all organs at late-stage infection (Fig 1D), fluorescence intensity and distribution varied (Fig 3). To investigate this discrepancy, further histological investigations were performed. Several organs exhibiting intense diffuse ZsG fluorescence—adrenal gland, Peyer’s patch, and reproductive tract—were shown by IHC to have both intravascular and extensive tissue NP antigen distribution (Fig 4A, top row). Viral antigen was detected in both the adrenal cortex and medulla; in macrophages in gut-associated lymphoid tissue; and extensively in the stroma of ovary, terminal oviduct, and uterus. However, organs with comparable viral RNA levels (i.e., kidney, lung, brain, and heart) consistently had relatively lower fluorescence intensity and/or distribution. In these organs, viral antigen was minimally detected in the tissue and primarily localized within intravascular leukocytes and rare endothelial cells (Fig 4A, bottom row). These 2 patterns of antigen distribution (tissue/intravascular or intravascular) were also observed by visualizing ZsG expression on formalin-fixed (>30 days fixed; imaged 2 months after slide preparation) unstained tissue using only a fluorescent microscope (Fig 4B). In the adrenal gland and liver, ZsG expression was both intravascular and in tissue, whereas in the kidney and lung, the majority of ZsG-expressing cells were present within the vasculature.

### Mononuclear phagocytic cells are early targets of CCHFV infection in Ifnar^-/-^ mice

**Fig 5:**
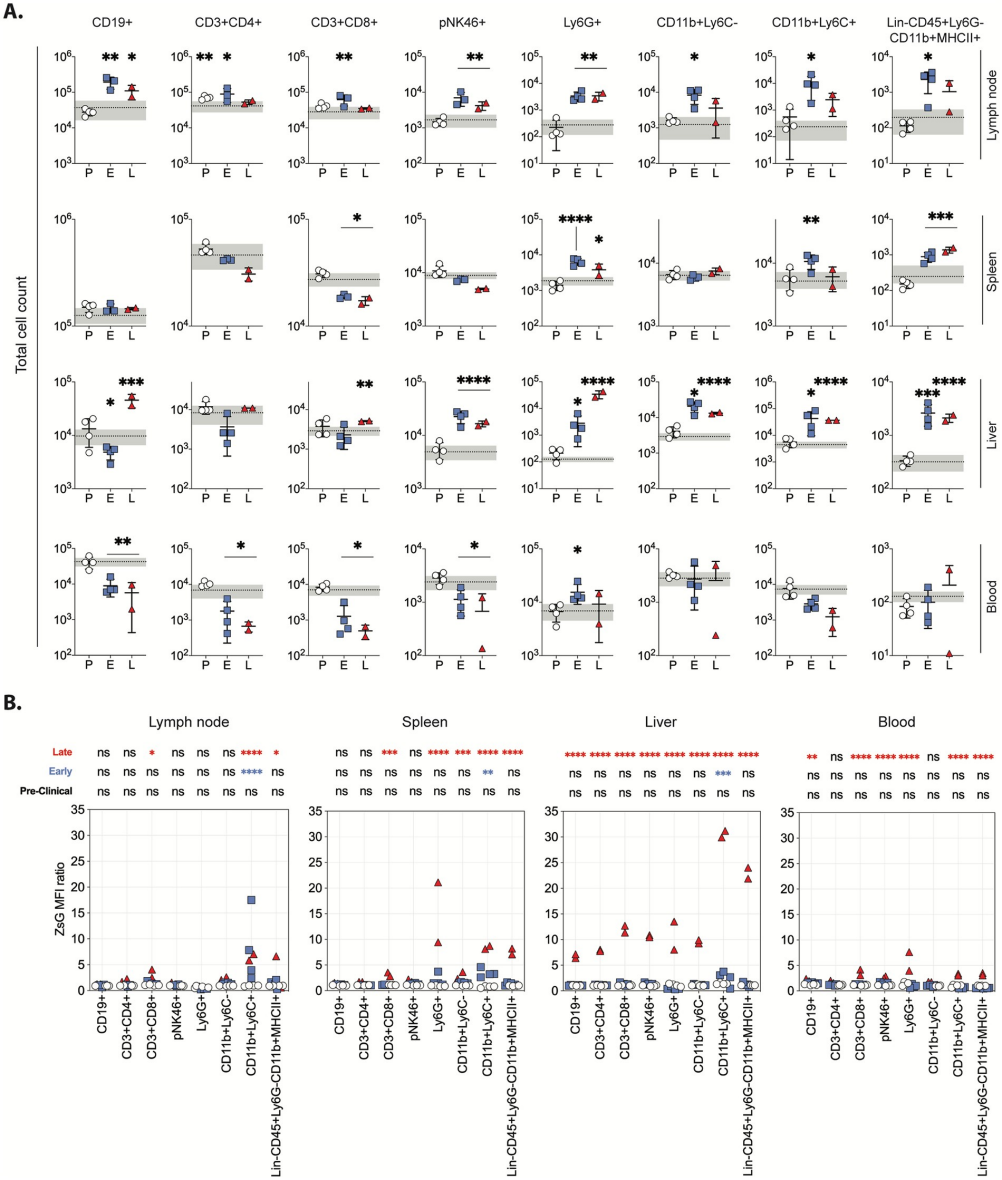
Cell population kinetics and susceptibility in mice infected with CCHFV/ZsG. (A) Differences in total absolute numbers of tissue-specific cells were determined for CCHFV/ZsG-infected mice classified as being in the pre-clinical (open circle; *n* = 4), early (blue square; *n* = 4), or late (red triangle; *n* = 2) stage of infection at time of euthanasia. Symbols represent individual animals, with error bars showing means and standard deviation. Dotted line represents the mean and the grey shading represents ±1 SD from mock-infected control animals (*n* = 6). (B) Graphs represent the ZsG mean fluorescent intensity (MFI) ratio of tissue-specific B cells (CD19+), T cells (CD3+CD4+ and CD3+CD8+), natural killer cells (NK; pNK46+), polymorphonuclear neutrophils (PMN; Ly6G+), immature monocytes/macrophages (CD11b+Ly6C-), activated monocyte/macrophages (CD11b+Ly6C+), and dendritic cells (DC; Lin-CD45+Ly6G-CD11b+MHCII+) over mock-infected controls (*n* = 6) for CCHFV/ZsG-infected mice classified as being in the pre-clinical (open circle; *n* = 4), early (blue square; *n* = 4), or late (red triangle; *n* = 2) stage of infection at time of euthanasia. Symbols represent individual animals. For both panels, significant change from levels in control animals is represented (if not stated, the difference was not significant). * *p < 0*.*05*, ** *p < 0*.*01*, *** *p < 0*.*001*, **** *p < 0*.*0001*. Data were analyzed by multiple t-test, with individual values indicated in a scatter dot plot (means ± SD).

In CCHF, the degree of leukopenia is correlated to severity of disease and fatal outcomes [15,16]. However, alterations in specific leukocyte populations over the course of disease are not clearly defined. In human CCHF, some reported cell population changes, like decreased peripheral blood lymphocyte (PBLs) and monocyte numbers, and increased neutrophil levels [17], are consistent, whereas others are contradictory. For example, levels of natural killer (NK) cells have been observed by some to decrease and by others not to change during CCHFV infection [18,19]. Thus, to better define cell population dynamics during infection, flow cytometry was performed on cells harvested from the axillary draining lymph node, spleen, liver, and blood of both wild-type CCHFV- and CCHFV/ZsG-infected mice euthanized during pre-clinical, early, and late-stage disease (Fig 5A, S2 Table). In pre-clinical mice, the only cell population with a significant change was CD4+ T cells (CD3+CD4+) from the lymph node, increasing over baseline from mock-infected animals (grey shading). In early-stage disease, significant increases were observed in all cell populations assessed in the lymph node. Levels of B cells (CD19+), NK cells (pNK46+), and neutrophils (Ly6G+) remained significantly elevated in late-stage disease, whereas levels of T cells (CD3+CD4+ and CD3+CD8+), and members of the mononuclear phagocyte system (monocytes/macrophages: CD11b+Ly6C- (immature) and CD11b+Ly6C+ (activated); dendritic cells [DC]: Lin-CD45+Ly6G-CD11b+MHCII+) all returned to baseline in late-stage disease animals. In the spleen, although levels of CD4+ T cells decreased with disease progression, the only significant change in lymphocyte levels was a reduction in CD8+ T cells in early- and late-stage disease animals. Levels of CD11b+Ly6C+ monocytes/macrophages, neutrophils, and DCs were all significantly elevated in spleens of animals in early-stage disease, with levels of the latter two also elevated during late-stage infection. In the liver, B cell levels decreased significantly in early-stage disease, although, along with CD8+ T cells, they then became elevated during late-stage infection. NK cell, CD11b+Ly6C+ and CD11b+Ly6C- macrophage, DC, and neutrophil levels were all significantly elevated in both early- and late-stage disease compared to mock-infected controls. In particular, neutrophil levels increased 22-fold and 275-fold from baseline levels in early- and late-stage disease, respectively. In the blood, significant lymphopenia was evident during early and late stage, with 10- to 20-fold reduction in B cells, CD4+ and CD8+ T cells, and NK cells. The only other significant change in blood cell counts was an increase in neutrophil numbers in early-stage disease.

In infected mice, we detected intravascular viral antigen predominantly in cells morphologically consistent with monocytes (Fig 4A), suggesting a role for members of the mononuclear phagocyte system (monocytes, macrophages, and DCs) in CCHFV infection. To investigate permissiveness of hematopoietic cell populations during infection, ZsG mean fluorescent intensity (MFI) was assessed in cells from mice at each stage of disease and compared to baseline levels in control mice to calculate a ZsG MFI ratio. Overall, ZsG detection at the cellular level directly correlated with ZsG fluorescence observed grossly. No significant shift in ZsG MFI ratio was seen in any of the pre-clinical mice compared to control mice (Fig 5B, S2 Table). ZsG was first detected in early-stage disease and was limited to activated monocyte/macrophage (CD11b+ Ly6C+) cells, with significant increases in mean ZsG MFI ratios detected in cells taken from the draining lymph node (3- to 20-fold over background), the spleen (3- to 5-fold increase), and the liver (3-fold increase). At late-stage infection, ZsG-expression in activated monocytes/macrophages remained slightly elevated in the lymph node, although significant signal was also observed in CD8+ T cells and DCs. In late-stage animals, splenic CD8+ T cells (2-fold increase), neutrophils (15-fold increase), Ly6C- (3-fold increase) and Ly6C+ (8-fold increase) monocytes/macrophages, and DCs (8-fold increase) all demonstrated significantly higher ZsG expression over baseline. All cell types collected from the liver in late-stage infection demonstrated significant shifts in ZsG MFI ratio, with the largest shifts observed in Ly6C+ (30-fold increase) monocytes/macrophages and DCs (22-fold increase).

### Cytokine expression levels indicate acute inflammation in CCHFV infection

**Fig 6:**
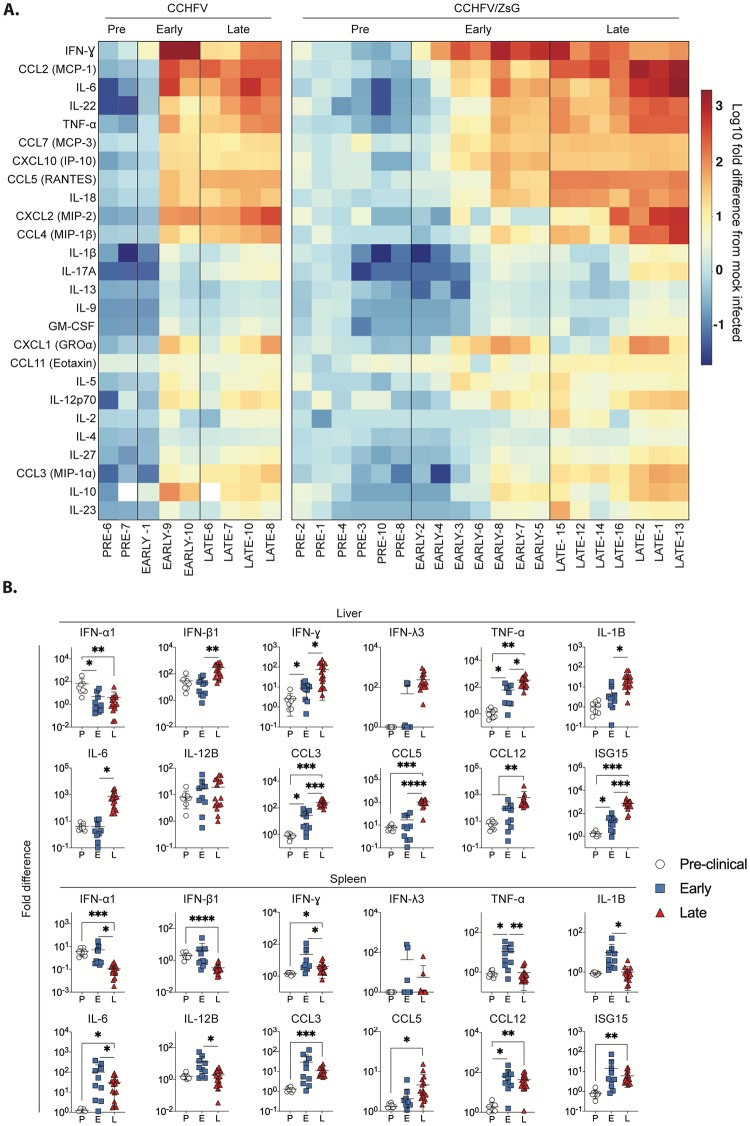
Differential cytokine expression in mice infected with either wild-type CCHFV or CCHFV/ZsG. (A) Cytokine and chemokine heatmap profiles represents the log_10_ fold differences in individual plasma cytokine levels or animals classified as being in either the pre-clinical (pre, *n* = 8), early (*n* = 10), or late (*n* = 11) stage of infection relative to those detected in control, mock-infected (*n* = 6) animals. Cytokine names are shown on the left, and individual animal designations on the bottom. CCL2, monocyte chemotactic protein 1 (MIP-1); CCL3, macrophage inflammatory protein 1α (MIP-1α); CCL4, macrophage inflammatory protein 1β (MIP-1 β); CCL5, regulated upon activation, normal T-cell expressed, and secreted (RANTES); CCL7, monocyte chemotactic protein 3 (MIP-3); CCL11, eosinophil chemotactic protein (eotaxin); CXCL1, chemokine (C-X-C motif) ligand-1 like; CXCL2, chemokine (C-X-C motif) ligand-2 like; macrophage inflammatory protein 2 (MIP-2); interferon-γ–induced protein 10 (IP-10); granulocyte-macrophage colony stimulating factor (GM-CSF); interferon γ (IFN-γ); interleukin (IL); tumor necrosis factor-α (TNF-α). (B) Graphs represent log_10_ fold differences (ΔΔCt) in select immune genes in the liver or spleen of mice infected with either CCHFV or CCHFV/ZsG, relative to mock-infected controls, in animals classified as in pre-clinical (P, white circles), early- (E, blue squares), or late- (L, red triangles) stage infection. Data were analyzed by multiple t-test, with individual values indicated in a scatter dot plot (means ± SD). * *p < 0*.*05*, ** *p < 0*.*01*, *** *p < 0*.*001*, **** *p < 0*.*0001*.

Severe CCHF is characterized by vascular dysfunction, with microvascular leakage resulting in hemorrhage and ultimately fulminant shock syndrome [16], similar to severe disease caused by Ebola, Marburg, and Lassa viruses [20]. However, the pathology associated with CCHF cannot be explained by viral-mediated tissue damage alone; IFN- and cytokine-mediated injury probably contribute to severe disease [21–23]. To analyze virus-associated cytokine levels in the plasma and tissues of Ifnar^-/-^ mice, Luminex- and PCR-based assays were performed, respectively. Cytokine levels for animals in pre-clinical, early, and late-stage disease were determined and compared to mock-infected control animals. Mice infected with wild-type CCHFV or CCHFV/ZsG demonstrated comparable cytokine level patterns at each clinical stage (Fig 6A, S3 Fig, S1 Data). To examine cytokine responses relative to disease progression, animals infected with wild-type CCHFV or CCHFV/ZsG were grouped together by clinical stage for analysis. The only significant changes in plasma cytokine levels in pre-clinical stage animals compared to controls were an increase in eotaxin (eosinophil chemotactic protein) and a reduction in IL-27 and IL-17A (Fig 6A, S3 Fig). In early-stage disease, mean levels of several cytokines were elevated, including significant increases in IP-10, IL-5, IL-22, IL-12p70, CXCL1, CCL7, and eotaxin. In late-stage disease, cytokines that remained significantly elevated compared to mock-infected controls, included IL-5, IL-22, IL-12p70, CCL7, and eotaxin. Furthermore, at this terminal stage of disease, IL-10, IL-1β, IL-2, IL-4, IL-9, IL-27, IFN-ɣ, GM-CSF, CCL5, TNF-α, CCL3, CCL2, IL-17A, and IL-18 were also found to be at significantly elevated levels.

In addition to investigating plasma levels, a subset of relevant cytokines was also assessed in the liver and spleen, tissues with high levels of virus and associated pathology in both mice and humans [7,24]. A panel of 12 analytes was quantified in groups of mice (infected with wild-type CCHFV or CCHFV/ZsG) sacrificed at 2, 4, and 5 dpi and compared to mock-infected control mice (Fig 6B, S1 Data). In the liver, levels of the cytokines examined tended to progressively increase with increasing disease severity and peaked during late-stage clinical disease, with significantly elevated levels of TNF-α, IL-1β, IL-6, CCL3, CCL5, CCL2, and ISG15 observed. Levels of IFN-β1, IFN-ɣ, and IFN-λ also all increased as disease progressed, but IFN-α1 levels significantly decreased along the same clinical time scale. In the spleen, cytokine levels tended to peak during early-stage infection and decrease during late-stage disease. Levels of TNF-α and CCL2 were significantly elevated from pre-clinical to early-stage infection, and IL-6, IL-12b, CCL3, CCL12, and ISG15 levels were significantly elevated from pre-clinical to late-stage infection. Both type-1 IFN levels were seen to significantly decrease in late-stage compared to pre-clinical animals.

## Discussion

The development of a reverse genetics system for CCHFV [25] has been instrumental for advanced pathogenesis studies [26,27], the development of high-throughput screening systems [11] and therapeutic candidates [28,29]. Here, we find that a recombinant CCHFV expressing ZsG is uniformly lethal in Ifnar^-/-^ mice, with a disease course comparable to that described for the parental virus (Fig 1). We have capitalized on the fluorescent expression to visualize virus distribution in tissues in situ (Fig 3), and to investigate cellular targets of infection in progressive stages of disease (Fig 5).

Sexual transmission of viral hemorrhagic fevers, as reported in Ebola [30–32], has significant public health implications. An unexpected finding was the extent of CCHFV distribution in reproductive tissues; the ovaries, terminal oviduct, and uterus all displayed intense ZsG expression correlating with extensive NP antigen IHC staining. Interestingly, despite high levels of viral antigen, pathology was minimal. This finding is similar to Ebola virus, which also infects reproductive tissues yet causes limited tissue immune responses and organ pathology in both male and female macaques [33]. Here, we were only able to examine female reproductive tissues, so whether these findings extend to male reproductive tissues and any implications for sexual transmission remain to be determined. Several reports focused on CCHF in pregnant women and outcomes in neonates [34–39], but to date, there is only one report of potential sexual transmission [40]. Our data support future investigations into reproductive infection, pathology, and implications for CCHFV transmission, as well as using both female and male models of disease for these and other pathogenesis studies.

In Ifnar^-/-^ mice, we detected CCHFV/ZsG-infected mononuclear phagocytic cells (MPC) early in disease. This is consistent with studies of other hemorrhagic fever viruses, including Ebola, Marburg, and Lassa [41–44]. The identification of CCHFV/ZsG fluorescence in MPCs, initially in draining lymph node, spleen and liver of Ifnar^**-/-**^ mice, coupled with the trafficking function of these cells, suggests a key role for these cell populations in systemic dissemination of the virus [41,45]. Macrophages and monocyte-derived DCs have been shown to be permissive to CCHFV infection in vitro [46,47]. While infection of DCs (Lin- CD45+ Ly6G- CD11b+ MHCII+) early in the course of disease was less apparent than that of monocytes (CD11b+ Ly6C+), a role for DCs in early infection cannot be excluded: our assays may not have been sensitive enough to detect them, or infected DCs may have been located in tissues that were not sampled. However, similar to in vitro data, DCs were clearly permissive to CCHFV infection in this model, and populations of infected DCs increased significantly in the liver in late-stage disease.

In these studies, we found a discrepancy between ZsG signal intensity in situ and virus detection by qRT-PCR. This may be due to our approach to imaging ZsG, which is largely limited to visualizing surface fluorescence. However, the disparate findings may also be due to tissue-specific distribution patterns of CCHFV. By IHC, we found 2 predominant patterns of antigen distribution: tissue/intravascular and intravascular. While levels of viral RNA were comparable between many organs; those with higher ZsG intensity had the former antigen pattern (e.g., liver), while those with lower relative intensity had the latter antigen pattern (e.g., kidney, lung). This suggests that high viral load by PCR is not necessarily indicative of parenchymal or stromal cell infection, and may reflect circulating virus instead. Together, the observed antigen staining patterns and flow cytometry target cell data suggest that following infection of mononuclear phagocytic cells, virus is widely disseminated intravascularly in most organs. However, in a subset of organs (i.e., liver, reproductive), the virus also has a tropism for parenchymal or stromal tissue cells. The parenchymal involvement in these organs explains changes in indicators of cellular injury and tissue dysfunction (i.e. elevated ALT, AST with liver damage), whereas endothelial staining in these and other organs is consistent with that observed in human infections, and contributes to hemorrhagic manifestations in severe disease.

In animal models of disease, identifying immunological parallels with and divergences from human disease is critical to support applicability of the model and guide translational studies. In addition to Ifnar^-/-^ mice, Stat-1^-/-^ [5], Rag2^-/-^ mice [10], and NSG-SGM3 humanized [8] mice have been reported to develop clinical signs following CCHFV infection. Ifnar^-/-^ mice are deficient in type I IFN signaling; Stat-1^-/-^ mice are deficient in type I, type II, and type III signaling; Rag2^-/-^ mice are unable to generate mature T or B lymphocytes; and humanized mice have a variety of potential alterations to the immune response based on deficiencies in the mouse background and humanization approach. Use of each of these strains presents inherent limitations for pathogenesis studies; the aforementioned alterations can affect immune cell populations [48] and associated innate and adaptive responses, which may be reflected in the level of associated cytokines/chemokines assessed. Largely, our findings were consistent with reports in other mouse models. We saw a peripheral blood lymphopenia correlating with increasing disease severity, similar to observations made in Stat-1^-/-^ [5] and Rag2^-/-^ mice [10], and increases in plasma levels of pro-inflammatory cytokines (IL-6 and IL-18) and chemoattractants (CCL2, CXCL1, and CXCL10) reported in the C57BL/6J (BL6) IFN-1-blockade disease model [10]. We did, however, note some variation in intensity of cytokine level changes than those reported in other mice. IL-1β increased less in Ifnar^-/-^ than in Stat-1^-/-^ and Rag2^-/-^ mice, whereas IFN-ɣ levels were much higher in Ifnar^-/-^ and STAT-1^-/-^ than in Rag2^-/-^ mice. These differences could be due to inherent immunological differences in the strains.

In addition to caveats in analyzing immune responses in immune-deficient mouse strains, direct comparisons between mouse and human immune responses are inherently challenging due to differences in a variety of response components (e.g., cell population subsets, receptors and signaling pathways) [49]. Despite the known challenges in drawing comparisons, we observed several parallels between the responses we recorded in Ifnar^-/-^ mice and reports of severe human disease. Ifnar^**-/-**^ mice demonstrated increased levels of proinflammatory cytokines (IL-1β, IL-6, CXCL10, TNF-α), and the anti-inflammatory cytokine IL-10, all of which have been associated with fatal outcomes in humans [50–52]. Increased levels of murine CXCL1 and CXCL2, functional homologs of human IL-8, correlate with reports of IL-8 as a diagnostic marker for disease severity in humans [52]. Finally, changes in clinical chemistry and CBC values reported in humans as important diagnostic markers for predicting disease severity, including elevated AST and ALT, elevated neutrophils, and lymphopenia [1,17,53,54], were also seen in Ifnar^**-/-**^ mice as disease severity increased.

CCHFV disease in humans is classified into 3 acute clinical phases, ending in either death or convalescence: an incubation period of 3–6 days, followed by a pre-hemorrhagic phase of 1–7 days, and a hemorrhagic phase of 1–10 days [55,56]. Human CCHF disease severity can range from mild to fatal. Disease in Ifnar^-/-^ mice infected with most CCHFV strains is severe [28,57,58], and, in the case of CCHFV strain IbAr10200, uniformly lethal even at low virus doses [6,7]. Examining data in mice grouped by clinical disease state revealed parallels to severe human disease. Here, animals were grouped according to clinical signs and viral load. The pre-clinical phase in mice largely resembled the incubation period in humans. Parallels to human disease seen in early-stage disease mice include: increased viral load; increased NK and activated macrophages cell numbers [19]; and increased levels of IL-10, IL-6, IFN-γ, CCL2, CCL5, and TNF-α [22,50,59] (also seen in late stage). Parallels seen in late-stage disease mice include: high viral loads (RNAemia) [60], elevated AST and ALT [61], elevated neutrophils [54], lymphopenia [1], and increases in IL-10, IL-6, IFN-γ, CCL2, CCL5, and TNF-α [22,50,59]. Identifying objective criteria to correlate disease course in mice to humans will allow improved translation of clinical and immunological indices (chemistry, biomarkers, cell- and antibody-mediated responses). Furthermore, identifying comparable points in disease progression may also support improved evaluation of treatments, in particular efficacy of regimens initiated at different stages of infection (i.e., prior to onset of clinical signs or during disease).

Here we find that CCHFV/ZsG infection in Ifnar^**-/-**^ mice is comparable in virulence, viral distribution, and pathology to infection with parental wild-type CCHFV. To our knowledge, this is the first report of a CCHFV expressing a fluorescent reporter protein in an animal model of disease. Furthermore, when scored based on disease progression, viral loads in blood and tissues, cell population dynamics, and corresponding cytokine responses over the course of disease reveal parallels between clinical phases of disease in Ifnar^**-/-**^ mice and those reported in humans, supporting broader applicability of mouse models. Based on demonstrated applicability of the recombinant virus, we were able to capitalize on viral-encoded fluorescence to image infected cells in situ, expanding our knowledge of tissue tropism and revealing tissue-specific patterns of viral distribution. Furthermore, our study is also the first report to identify early hematopoietic cellular targets of infection via flow cytometry. Together, these data provide key insights into both early and late events in CCHF pathogenesis, expanding our knowledge of cellular and tissue targets of infection.

## Materials and methods

### Biosafety

All CCHFV infections were performed in biosafety level 4 facilities at the Centers for Disease Control and Prevention (Atlanta, GA, USA). Experiments involving cDNA-encoding viral sequences were performed in accordance with approved Institutional Biosafety Committee protocols.

### Ethics statement

All animal procedures were approved by the CDC Institutional Animal Care and Use Committee (IACUC protocol 2797SPEMOUC) and conducted in accordance with the *Guide for the Care and Use of Laboratory Animals* [62]. The CDC is fully accredited by the AAALAC-International.

### Virus rescue and titrations

Rescue of recombinant CCHFV (based on strain IbAr10200; GenBank KJ648914, KJ648915, and KJ648913) and recombinant CCHFV/ZsG expressing the fluorescent protein ZsG were performed as previously described [11,25]. Viral titers were calculated as TCID_50_ [63], and were determined in BSR-T7/5 cells by indirect immunofluorescence [26] or ZsG fluorescence. All recombinant virus work was approved by the CDC Institutional Biosafety Committee. All viral stocks were verified by next-generation sequencing and confirmed to be mycoplasma-free.

### Mouse infections

Female B6.129S2-*Ifnar1*^*tm1Agt*^/Mmjax mice (MMRRC 032045-JAX; 7–8 weeks of age) were inoculated subcutaneously in the inter-scapular region with a target dose of 100 TCID_50_ of recombinant wild-type CCHFV or CCHFV/ZsG, or with DMEM (mock-infected controls). Mice were housed in a climate-controlled laboratory with a 12 h day/night cycle; provided sterilized commercially available mouse chow and water *ad libitum*; and group-housed on autoclaved corn cob bedding (Bed-o’Cobs ¼”, Anderson Lab Bedding) with cotton nestlets, in an isolator-caging system (Thoren Caging, Inc., Hazleton, PA, USA) with a HEPA-filtered inlet and exhaust air supply. Mice were humanely euthanized with isoflurane vapor followed by cervical dislocation at the indicated time points, or when clinical illness scores based on piloerection, behavior (e.g., reluctance to leave nest), activity level, neurological signs (ataxia, tremors, paresis/paralysis), dehydration, dyspnea, and/or weight loss (>20% from baseline at -1 dpi) indicated that the animal was in distress or in the terminal stages of disease.

### Fluorescent imaging

Fluorescence was visualized and imaged in situ using a Canon PowerShot G12 camera in conjunction with a Dark Reader camera filter (#AF580), Dark Reader Spot Lamp (#SL 10S), Dark Reader Hand Lamp (#HL34T), and Dark Reader glasses (#AG16), all from Clare Chemical Research (Dolores, CO). Images of ZsG fluorescence in fixed tissue mounted on slides was performed using an EVOS cell imaging system (Thermo Fisher Scientific).

### Clinical chemistry

Whole blood samples, when available, were collected in lithium heparin and analyzed on Piccolo Xpress chemistry analyzers (General Chemistry 13 Panel, Abaxis) within 1 h of collection.

### Quantitative RT-PCR

RNA was extracted from 25 μL whole blood (in LiH or EDTA) and homogenized tissue (~3 mm^3^ section) using the MagMAX-96 Total RNA Isolation Kit (Thermo Fisher Scientific) on a 96-well ABI MagMAX extraction platform, with an additional DNase-I treatment step according to manufacturer’s instructions, and was eluted in 75 μL MagMAX elution buffer. CCHFV RNA was quantified using a qRT-PCR assay targeting NP (forward primer, ATGAACAGGTGGTTTGAAGAGTT; reverse primer, TGGCACTGGCCATCTGA; probe, 6FAM-TGTCCAAATTGGGAACACTCTCGCA-BBQ; all TIB Molbiol) and standardized to 18S RNA levels (Thermo Fisher Scientific) with a SuperScript III Platinum One-Step qRT-PCR kit (Thermo Fisher Scientific) according to manufacturer’s instructions. CCHFV S genome copy numbers per μL of extracted RNA elution were calculated using a standard curve of in vitro-transcribed RNA of known copy numbers. Assays for murine IFN-α1, IFN-β1, IFN-ɣ, IFN-λ3, TNF-α, IL-1β, IL-6, IL-12b, CCL3 (MIP-1α), CCL5 (RANTES), CCL12 (MCP-5), and ISG15 were obtained from Thermo Fisher Scientific, and run with a SuperScript III Platinum One-Step qRT-PCR Kit (Thermo Fisher Scientific) according to manufacturer’s instructions. Cytokine expression levels in liver and spleen tissue from infected mice sacrificed at 2, 4, and 5 dpi relative to those from mock-infected (DMEM only) control mice sacrificed at the same day post challenge were calculated using ΔΔCt (18S RNA levels as the control).

### Histology and IHC

Tissue specimens were fixed in 10% neutral buffered formalin and gamma-irradiated (5 × 10^6^ rad). Tissues were routinely processed for paraffin embedding, sectioning, and staining with hematoxylin and eosin. For IHC assays, slides were stained with rabbit anti-CCHFV NP pAb (IBT Bioservices, #04–0011) diluted 1:1000, as previously described [8].

### Tissue processing for flow cytometry

Whole blood collected in EDTA was lysed with RBC lysis buffer, and the remaining cells were pelleted, resuspended in PBS, and directly processed for flow cytometry. A single-cell suspension of the axillary lymph node was made by dissociating the tissue in PBS using an Eppendorf tube with a plastic pestle before passing it through a 70 μM filter-top cell strainer snap-cap tube (Corning). Cells were pelleted and resuspended in PBS for flow cytometry. Whole liver and spleen (minus small sections removed for qRT-PCR) were disrupted by pushing tissue through a 70 μM cell strainer (Sigma) into a 50 mL conical tube and washing the strainer with either PBS (liver) or RMPI (spleen). Cells were pelleted by centrifugation, after which splenocytes were subjected to RBC lysis, washed with RPMI, and counted; 2 × 10^6^ cells were processed for flow cytometry. Liver cell pellets were suspended in PBS and layered onto Histopaque 1077 (Sigma-Aldrich) and separated by centrifugation at 500 × g for 30 min (no brake). The interface was collected and washed in PBS, and cells were processed for flow cytometry.

### Flow cytometry

Cells were washed in PBS, incubated in Near IR live/dead stain (1:500 in PBS) for 10 min at room temperature, and washed in flow buffer (PBS with 2% FCS). Surface stains were added (see S3 Table for panel details), and cells were incubated for 30 min at room temperature, then washed twice in flow buffer. Cells were then suspended in Cytofix/Cytoperm (BD) for 20 min at room temperature. After 2 washes in BD perm/wash, cells were incubated with intracellular stains for 45 min at room temperature. Following additional washes, cells were suspended in PBS and events were collected on a Stratedigm S1000EXi (gating strategy is shown in S4 Fig). Compensation controls were generated using OneComp eBeads (eBioscience).

### Luminex cytokine expression

Plasma samples were gamma-irradiated (5.0 × 10^6^ rads), then analyzed using a murine cytokine magnetic 26-plex ProcartaPlex Panel 1 (25 μL of sample; Thermo Fisher Scientific, EPXR260-26088-901) per manufacturer’s instructions using a 2 h incubation, and read on a Luminex 200 platform. Cytokines analyzed were CCL2 (MCP-1), CCL3 (MIP-1α), CCL4 (MIP-1β), CCL5 (RANTES), CCL7 (MCP-3), CCL11 (eotaxin), CXCL1 (GRO-α), CXCL2 (MIP-2), CXCL10 (IP-10), GM-CSF, IL-1β, IL-2, IL-4, IL-6, IL-9, IL-10, IL-12p70, IL-13, IL-15, IL-17A, IL-18, IL-22, IL-23, IL-27, IFN-ɣ, and TNF-α.

### Graphing and statistical analyses

Survival statistics were calculated using the Mantel-Cox test. If animals were found dead on early morning checks, the intermediate time-point was used for statistical analyses (i.e., if found dead on the morning of the 5th dpi, statistical time of death was 4.5 dpi). Weight statistics were calculated using multiple t-tests with Holm-Siddack’s multiple comparison test. Clinical chemistry and ZsG MFI ratio statistics were calculated using two-way ANOVA with Tukey’s multiple comparison test. Absolute cell population numbers, Luminex cytokine expression absolute values, and immune response qRT-PCR ΔΔCt fold-change data were analyzed by multiple t-test. All analyses and graphs were performed using GraphPad Prism v8.0 software. Luminex heatmaps were prepared using BioVinci (v1.1.4, BioTuring).

## Supporting information

S1 Fig Representative images of NP immunostaining demonstrating increasing antigen expression with disease progression in the adrenal gland, kidney, lung, ovary, uterus, and Peyer’s patch of Ifnar^-/-^ mice inoculated subcutaneously with 100 TCID_50_ of CCHFV/ZsG. All tissues lack detectable immunostaining of NP antigen in the preclinical stage of disease (left column). Immunostaining progressively increases in all organs from early- (middle column) to late- (right column) stage disease, with antigen localization to epithelial cells and vasculature in the adrenal gland, intravascular leukocytes and rare interstitial cells in the kidney and lung, stromal cells in the ovary, endometrial and myometrial cells in the uterus, and in primarily mononuclear phagocytic cells of intestinal Peyer’s patches.
(TIF)

S2 Fig Representative images of NP immunostaining demonstrating increasing antigen expression with disease progression in the adrenal gland, kidney, lung, Peyer’s patch, and lymph node of Ifnar^-/-^ mice inoculated subcutaneously with 100 TCID_50_ of wild-type CCHFV. Immunostaining patterns are similar for wild-type CCHFV-inoculated mice as for CCHFV/ZsG-inoculated mice shown in S1 Fig, with lack of NP immunostaining in the pre-clinical stage of disease (left panel), and progressive increase in staining from early- (middle column) to late- (right column) stage disease for all organs. All lack immunostaining of NP antigen in the pre-clinical stage of disease (left column). Immunostaining progressively increases in all organs from early- (middle column) to late- (right column) stage disease, with antigen localization to epithelial cells and vasculature in the adrenal gland, intravascular leukocytes and rare interstitial cells in the kidney and lung, and primarily mononuclear phagocytic cells in lymph nodes and intestinal Peyer’s patches.
(TIF)

S3 Fig Plasma cytokine and chemokine profiles from Ifnar^-/-^ mice classified as either in pre-clinical (white circles; *n* = 10), early- (blue squares; *n* = 7), or late-stage (red triangles; *n* = 12) disease following subcutaneous inoculation with 100 TCID_50_ of either CCHFV or CCHFV/ZsG. Control animals (black circles, *n* = 6) were mock-infected with DMEM. Data were analyzed by multiple t-test, with individual values indicated in a scatter dot plot (means ± SD). * *p < 0*.*05*, ** *p < 0*.*01*, *** *p < 0*.*001*, **** *p < 0*.*0001*. CCL2, monocyte chemotactic protein 1 (MIP-1); CCL3, macrophage inflammatory protein 1α (MIP-1α); CCL4, macrophage inflammatory protein 1β (MIP-1 β); CCL5, regulated upon activation, normal T-cell expressed, and secreted (RANTES); CCL7, monocyte chemotactic protein 3 (MIP-3); CCL11, eosinophil chemotactic protein (eotaxin); CXCL1, chemokine (C-X-C motif) ligand-1 like; CXCL2, chemokine (C-X-C motif) ligand-2 like; macrophage inflammatory protein 2 (MIP-2); interferon-γ–induced protein 10 (IP-10); granulocyte-macrophage colony stimulating factor (GM-CSF); interferon-γ (IFN-γ); interleukin (IL); tumor necrosis factor-α (TNF-α).
(TIF)

S4 Fig Gating strategy for (A) lymphocyte panel and (B) antigen-presenting cell (APC) panel. The strategy is demonstrated on blood samples and was also applied to lymph node, spleen, and liver samples. Normal healthy mice were used to illustrate the gating strategy.
(TIF)

S1 Table Clinical classification and scoring of CCHFV and CCHFV/ZsG-infected animals. (DOCX)

S2 Table Flow cytometry values for total cell number and MFI ratio. (DOCX)

S3 Table Antibodies used in flow cytometric analyses. All antibodies from Biolegend (San Diego, CA, USA).
(DOCX)

S1 Data Cytokine and chemokine values in CCHFV- or CCHFV/ZsG-infected Ifnar^-/-^ mice (100 TCID_50_ SC). Mice were grouped based on viral RNA levels and weight loss as pre-clinical (*n* = 10), early- (*n* = 7), or late-stage (*n* = 12) disease. Control animals (*n* = 6) were mock infected with DMEM. **Luminex absolute values (Tab 1) and relative values (Tab 2)**. Luminex values in pg/mL for 26 plasma cytokine and chemokine levels. **Fold change values (ΔΔCt) in Liver (Tab 3) and Spleen RNA (Tab 4)**. RNA quantification of 12 liver and spleen cytokines from CCHFV- or CCHFV/ZsG-infected Ifnar^-/-^ mice. CCL2, monocyte chemotactic protein 1 (MIP-1); CCL3, macrophage inflammatory protein 1α (MIP-1α); CCL4, macrophage inflammatory protein 1β (MIP-1 β); CCL5, regulated upon activation, normal T-cell expressed, and secreted (RANTES); CCL7, monocyte chemotactic protein 3 (MIP-3); CCL11, eosinophil chemotactic protein (eotaxin); CXCL1, chemokine (C-X-C motif) ligand-1 like; CXCL2, chemokine (C-X-C motif) ligand-2 like; macrophage inflammatory protein 2 (MIP-2); interferon-γ–induced protein 10 (IP-10); granulocyte-macrophage colony stimulating factor (GM-CSF); interferon γ (IFN-γ); interleukin (IL); tumor necrosis factor-α (TNF-α); interferon (IFN); CCL12, monocyte chemotactic protein 5 (MCP-5); interferon stimulated gene 15 (ISG15).
(XLSX)
