# Supplementary figures and images for: Fluorescent Crimean-Congo hemorrhagic fever virus illuminates tissue tropism patterns and identifies early mononuclear phagocytic cell targets in Ifnar-/- mice
Source: PLoS Pathog. 2019 Dec 2;15(12):e1008183. doi: 10.1371/journal.ppat.1008183 (PMC6984736; doi:10.1371/journal.ppat.1008183)

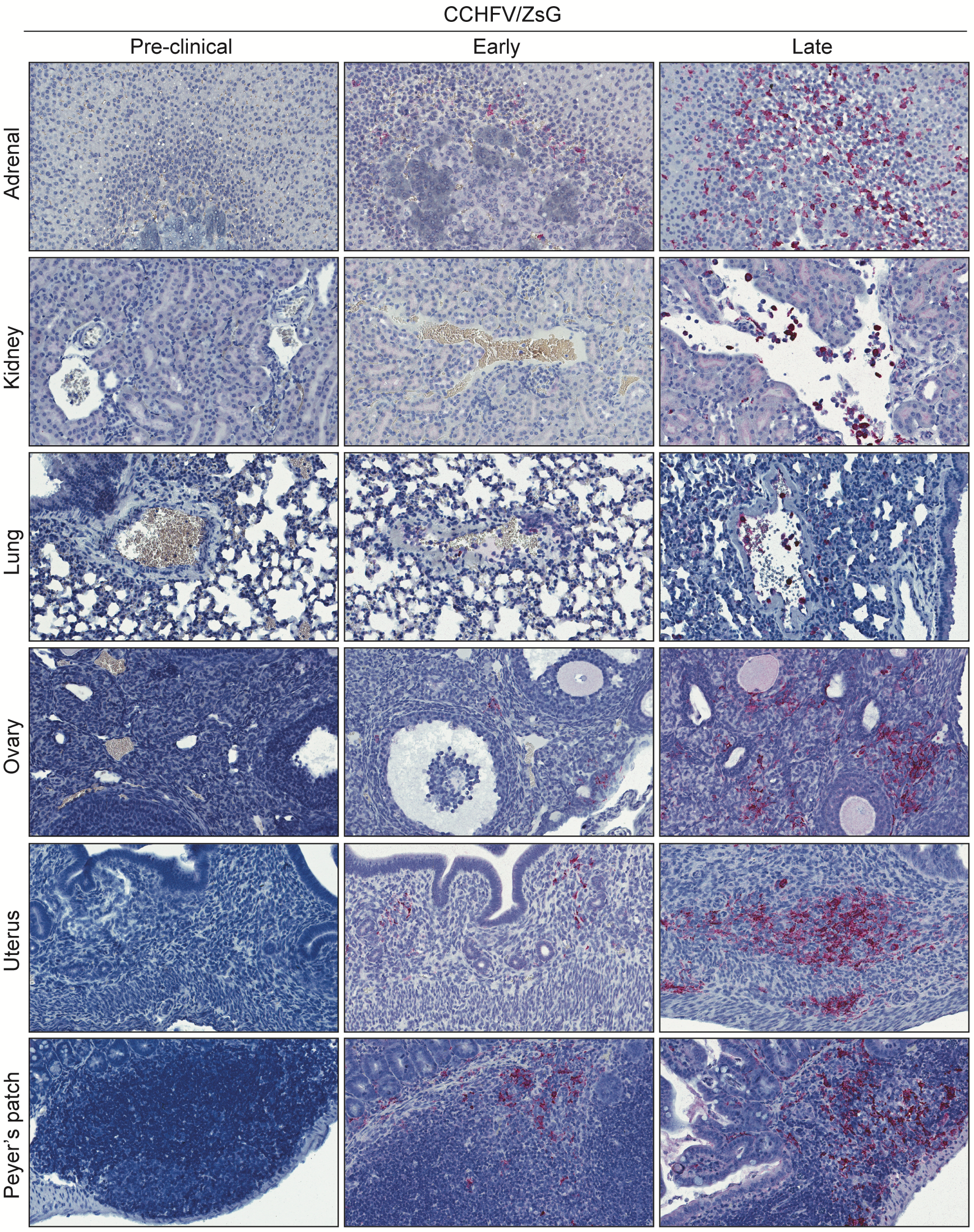

Supplement: S1 Fig — All tissues lack detectable immunostaining of NP antigen in the preclinical stage of disease (left column). Immunostaining progressively increases in all organs from early- (middle column) to late- (right column) stage disease, with antigen localization to epithelial cells and vasculature in the adrenal gland, intravascular leukocytes and rare interstitial cells in the kidney and lung, stromal cells in the ovary, endometrial and myometrial cells in the uterus, and in primarily mononuclear phagocytic cells of intestinal Peyer’s patches. (TIF) [file ppat.1008183.s001.tif]

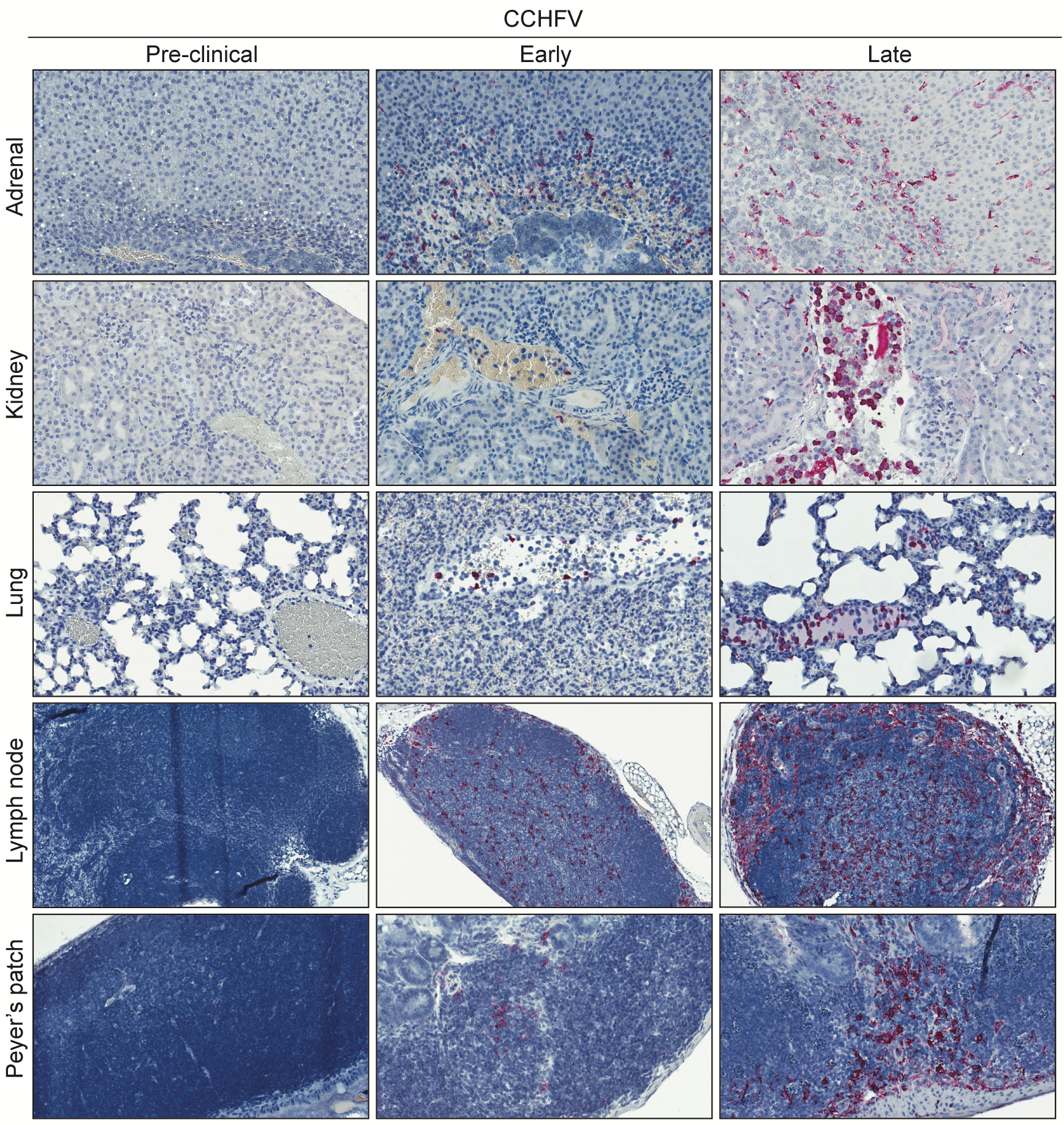

Supplement: S2 Fig — Immunostaining patterns are similar for wild-type CCHFV-inoculated mice as for CCHFV/ZsG-inoculated mice shown in S1 Fig, with lack of NP immunostaining in the pre-clinical stage of disease (left panel), and progressive increase in staining from early- (middle column) to late- (right column) stage disease for all organs. All lack immunostaining of NP antigen in the pre-clinical stage of disease (left column). Immunostaining progressively increases in all organs from early- (middle column) to late- (right column) stage disease, with antigen localization to epithelial cells and vasculature in the adrenal gland, intravascular leukocytes and rare interstitial cells in the kidney and lung, and primarily mononuclear phagocytic cells in lymph nodes and intestinal Peyer’s patches. (TIF) [file ppat.1008183.s002.tif]

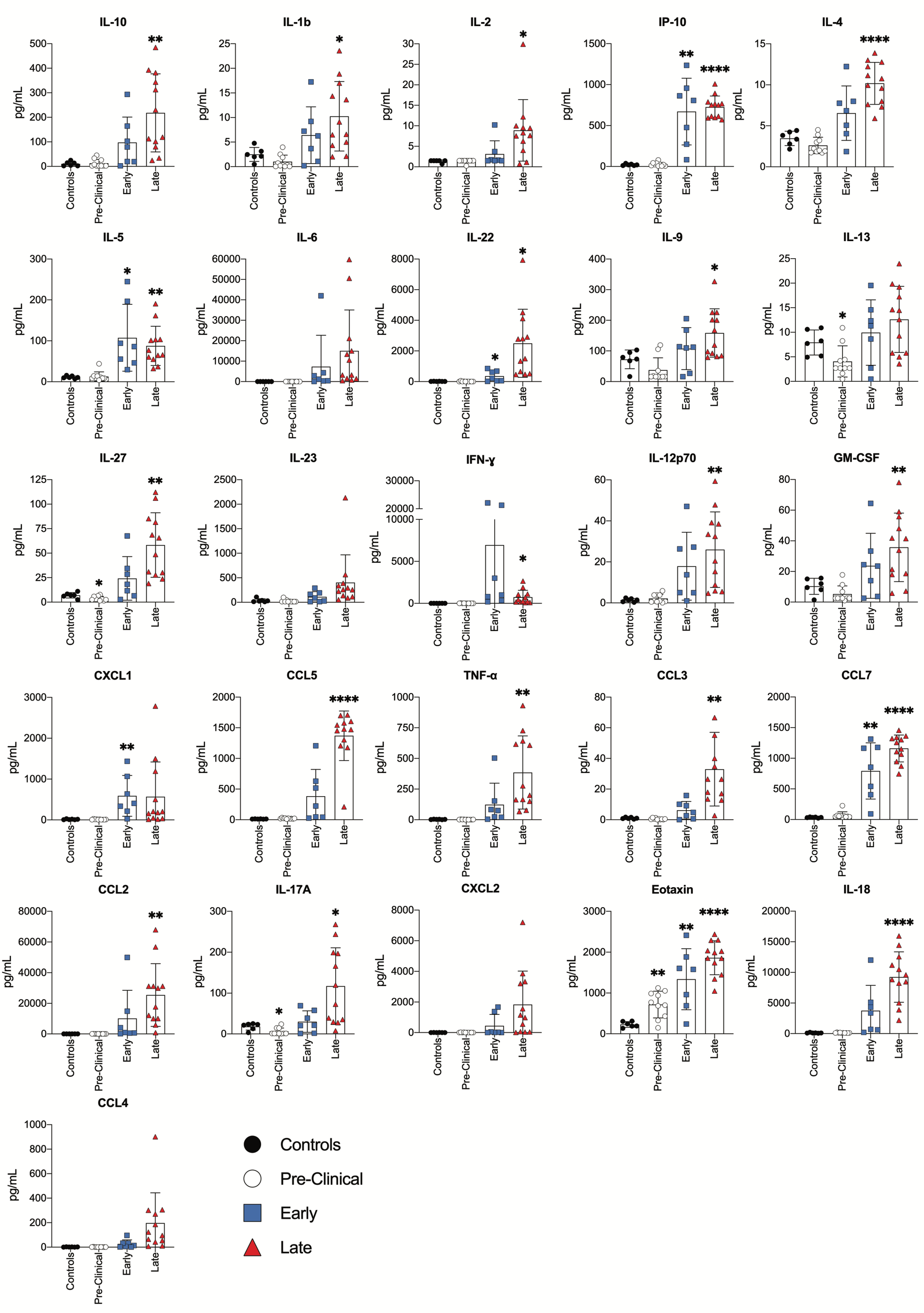

Supplement: S3 Fig — Control animals (black circles, n = 6) were mock-infected with DMEM. Data were analyzed by multiple t-test, with individual values indicated in a scatter dot plot (means ± SD). * p < 0.05, ** p < 0.01, *** p < 0.001, **** p < 0.0001. CCL2, monocyte chemotactic protein 1 (MIP-1); CCL3, macrophage inflammatory protein 1α (MIP-1α); CCL4, macrophage inflammatory protein 1β (MIP-1 β); CCL5, regulated upon activation, normal T-cell expressed, and secreted (RANTES); CCL7, monocyte chemotactic protein 3 (MIP-3); CCL11, eosinophil chemotactic protein (eotaxin); CXCL1, chemokine (C-X-C motif) ligand-1 like; CXCL2, chemokine (C-X-C motif) ligand-2 like; macrophage inflammatory protein 2 (MIP-2); interferon-γ–induced protein 10 (IP-10); granulocyte-macrophage colony stimulating factor (GM-CSF); interferon-γ (IFN-γ); interleukin (IL); tumor necrosis factor-α (TNF-α). (TIF) [file ppat.1008183.s003.tif]

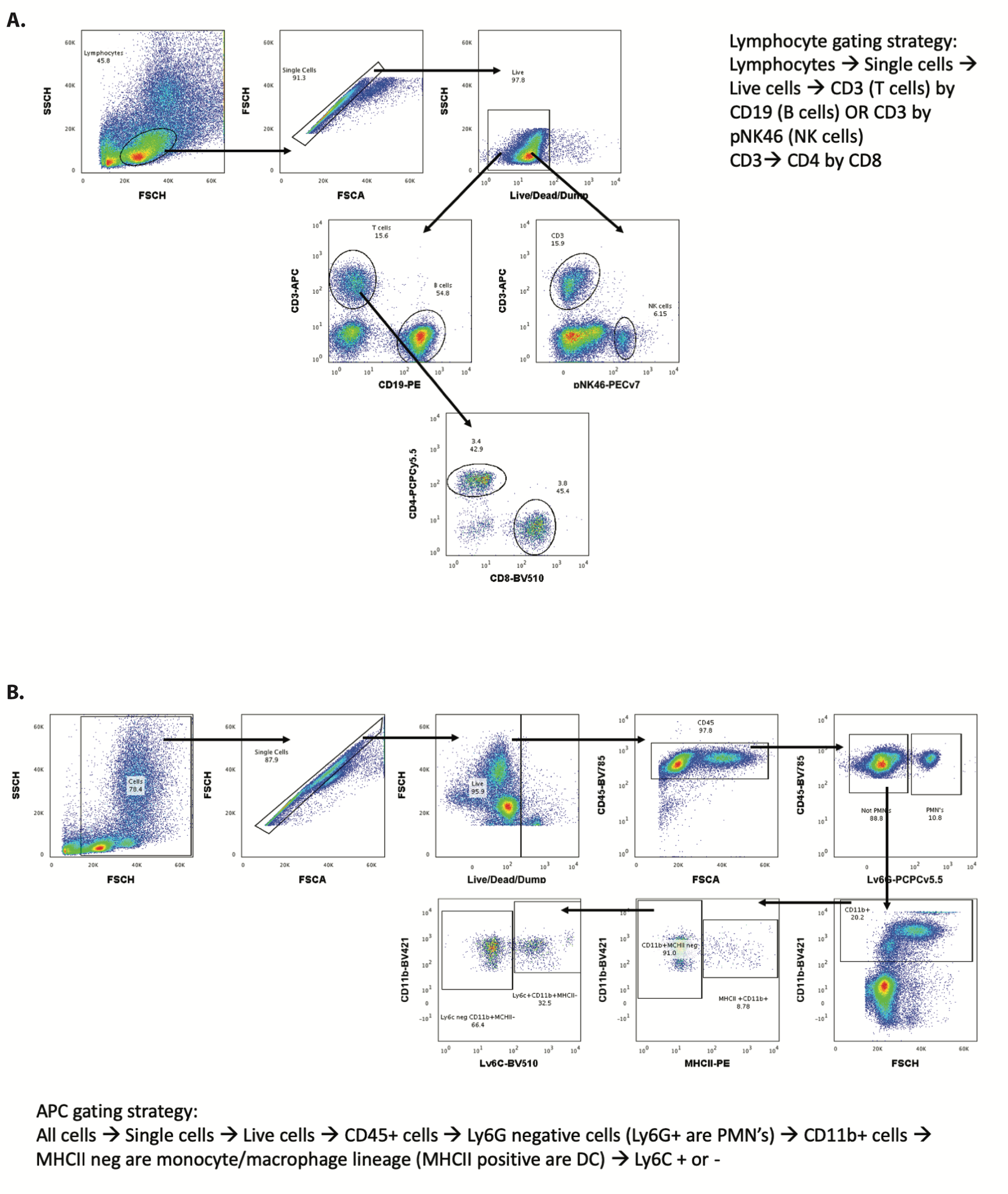

Supplement: S4 Fig — The strategy is demonstrated on blood samples and was also applied to lymph node, spleen, and liver samples. Normal healthy mice were used to illustrate the gating strategy. (TIF) [file ppat.1008183.s004.tif]
