# Supplementary material for: Fluorescent Crimean-Congo hemorrhagic fever virus illuminates tissue tropism patterns and identifies early mononuclear phagocytic cell targets in Ifnar-/- mice
Source: PLoS Pathog. 2019 Dec 2;15(12):e1008183. doi: 10.1371/journal.ppat.1008183 (PMC6984736; doi:10.1371/journal.ppat.1008183)
Supplement: S1 Table — (DOCX) [file ppat.1008183.s005.docx]

**S1 Table.** Clinical classification and scoring of CCHFV and CCHFV/ZsG-infected animals

| **Classification** | **ID** | **Virus** | **DPI** | **Weight loss at euthanasia***  **(*score*)** | **CCHFV S genome copies/µL (score)** | | | **Total Score** |
| --- | --- | --- | --- | --- | --- | --- | --- | --- |
|  |  |  |  |  | **Liver** | **Spleen** | **Blood** |  |
| **Pre-clinical** | Pre-1 | ZsG | 2 | -2% (1) | 1.6 × 10^4^ (1) | 1.3 × 10^4^ (1) | ND | **3** |
|  | Pre-2 | ZsG | 2 | 3% (0) | 2.2 × 10^3^ (1) | 8.6 × 10^3^ (1) | ND | **2** |
|  | Pre-3 | ZsG | 2 | 6% (0) | 2.9 × 10^5^ (2) | 4.8 × 10^3^ (1) | ND | **3** |
|  | Pre-4 | ZsG | 2 | 1% (0) | ND | ND | ND | **0** |
|  | Pre-5 | WT | 2 | -2% (1) | ND | ND | ND | **1** |
|  | Pre-6 | WT | 2 | 4% (0) | ND | 6.0 × 10^4^ (1) | ND | **1** |
|  | Pre-7 | ZsG | 2 | -1% (1) | ND | ND | ND | **1** |
|  | Pre-8 | ZsG | 2 | 3% (0) | 1.4 × 10^4^ (1) | ND | ND | **1** |
| **Early** | Early-1 | WT | 2 | -9% (2) | 2.2 × 10^5^ (2) | 3.0 × 10^4^ (1) | ND | **6** |
|  | Early-2 | ZsG | 2 | -1% (1) | 9.9 × 10^5^ (2) | 2.8 × 10^5^ (2) | ND | **5** |
|  | Early-3 | ZsG | 2 | -3% (1) | 1.1 × 10^5^ (2) | 1.8 × 10^6^ (2) | 2.5 × 10^1^ (1) | **6** |
|  | Early-4 | ZsG | 2 | -2% (1) | 1.1 × 10^6^ (2) | 1.6 × 10^5^ (2) | ND | **5** |
|  | Early-5 | ZsG | 4 | -10% (2) | 1.6 × 10^7^ (3) | 3.2 × 10^7^ (3) | 2.0 × 10^7^ (3) | **11** |
|  | Early-6 | ZsG | 4 | -3% (1) | 3.6 × 10^6^ (2) | 3.0 × 10^6^ (2) | 8.5 × 10^7^ (3) | **8** |
|  | Early-7 | ZsG | 4 | -9% (2) | 3.4 × 10^7^ (3) | 2.8 × 10^7^ (3) | 5.9 × 10^7^ (3) | **11** |
|  | Early-8 | ZsG | 5 | -9% (2) | 6.3 × 10^6^ (2) | 5.5 × 10^7^ (3) | 2.0 × 10^7^ (3) | **10** |
|  | Early-9 | WT | 4 | -10% (2) | 2.2 × 10^7^ (3) | 2.8 × 10^7^ (3) | 3.2 × 10^7^ (3) | **11** |
|  | Early-10 | WT | 4 | -10% (2) | 3.8 × 10^7^ (3) | 3.0 × 10^7^ (3) | 8.3 × 10^7^ (3) | **11** |
| **Late** | Late-1 | ZsG | 4 | -15% (4) | 1.7 × 10^8^ (3) | 5.6 × 10^7^ (3) | 3.9 × 10^9^ (4) | **14** |
|  | Late-2 | ZsG | 5 | -12% (4) | 8.1 × 10^7^ (3) | 2.2 × 10^7^ (3) | 1.4 × 10^9^ (4) | **14** |
|  | Late-3 | ZsG | 5^†^ | -14% (4) | 7.3 × 10^7^ (3) | 2.7 × 10^8^ (3) | NS | NA |
|  | Late-4 | ZsG | 5^†^ | -10% (2) | 8.1 × 10^7^ (3) | 1.7 × 10^7^ (3) | NS | NA |
|  | Late-5 | WT | 5 | -17% (6) | 5.0 × 10^8^ (3) | 2.3 × 10^9^ (4) | 2.5 × 10^10^ (4) | **17** |
|  | Late-6 | WT | 5 | -19% (6) | 2.3 × 10^9^ (4) | 6.2 × 10^8^ (3) | 2.3 × 10^10^ (4) | **17** |
|  | Late-7 | WT | 5 | -22% (8) | 1.9 × 10^9^ (4) | 1.2 × 10^9^ (4) | 4.8 × 10^9^ (4) | **20** |
|  | Late-8 | WT | 5 | -15% (4) | 2.4 × 10^9^ (4) | 1.8 × 10^9^ (4) | 1.0 × 10^10^ (4) | **16** |
|  | Late-9 | WT | 5^†^ | -12% (4) | 3.1 × 10^9^ (4) | 1.4 × 10^9^ (4) | NS | NA |
|  | Late-10 | WT | 4 | -20% (8) | 1.2 × 10^8^ (3) | 8.2 × 10^7^ (3) | 3.4 × 10^9^ (4) | **18** |
|  | Late-11 | ZsG | 5^†^ | -14% (4) | 4.6 × 10^8^ (3) | 6.5 × 10^8^ (3) | NS | NA |
|  | Late-12 | ZsG | 6 | -19% (6) | 3.2 × 10^8^ (3) | 2.5 × 10^8^ (3) | 6.3 × 10^8^ (3) | **15** |
|  | Late-13 | ZsG | 5 | -19% (6) | 1.7 × 10^9^ (4) | 1.0 × 10^9^ (4) | 7.2 × 10^9^ (4) | **18** |
|  | Late-14 | ZsG | 6 | -19% (6) | 3.0 × 10^9^ (4) | 2.8 × 10^9^ (4) | 1.8 × 10^8^ (3) | **17** |
|  | Late-15 | ZsG | 6 | -17% (6) | 5.6 × 10^8^ (3) | 6.5 × 10^8^ (3) | 2.2 × 10^8^ (3) | **15** |
|  | Late-16 | ZsG | 4 | -18% (6) | 1.1 × 10^8^ (3) | 4.1 × 10^7^ (3) | 2.2 × 10^8^ (3) | **15** |

^†^Found dead in cage. *Last weight obtained prior to euthanasia or found dead. DPI, days post infection; NA, not applicable: any animals found dead in cage were considered late-stage disease regardless of combined score. ND, not detected; NS, samples not available; WT, recombinant wild-type CCHFV; ZsG, recombinant CCHFV/ZsG. Weight loss scoring criteria: 0 to -5% = 1; -6 to -10% = 2; -11 to 15%= 4; -16 to 20% = 6; > -20% = 8. Viral load scoring criteria: ≤10^5^ = 1; > 10^5^ – 10^7^ = 2; >10^7^ – 10^9^ = 3; >10^9^ – 10^11^ = 4. Classification scores: Pre-clinical < 5; Early = 5 to 12; Late > 12. Weight loss scoring was allocated larger values than viral load scoring (i.e., 1, 2, 4, 6, and 8 vs. 1, 2, 3, and 4), as the degree of weight loss was found to be more reflective of clinically observed disease severity.
