# Supplementary material for: Fluorescent Crimean-Congo hemorrhagic fever virus illuminates tissue tropism patterns and identifies early mononuclear phagocytic cell targets in Ifnar-/- mice
Source: PLoS Pathog. 2019 Dec 2;15(12):e1008183. doi: 10.1371/journal.ppat.1008183 (PMC6984736; doi:10.1371/journal.ppat.1008183)
Supplement: S2 Table — (DOCX) [file ppat.1008183.s006.docx]

**S2 Table.** Flow cytometry values for total cell number and MFI ratio.

|  |  | **Lymph node** | |  | **Spleen** | |  | **Liver** | |  | **Blood** | |
| --- | --- | --- | --- | --- | --- | --- | --- | --- | --- | --- | --- | --- |
|  |  | **Mean cell number (range)** | **Mean MFI (range)** |  | **Mean cell number (range)** | **Mean MFI (range)** |  | **Mean cell number (range)** | **Mean MFI (range)** |  | **Mean cell number (range)** | **Mean MFI (range)** |
| **CD19+** | Cont. | 37154 (73333-16086) | 10.2 (10.9-9.8) |  | 125819 (154876-94961) | 9.5 (11.0-8.5) |  | 9657 (13524-6117) | 14.2 (17.6-12.0) |  | 42645 (61097-31816) | 11.3 (16.2-6.2) |
|  | Pre | 27794 (35042-20221) | 9.6 (9.7-9.6) |  | 149886 (162448-127110) | 8.6 (9.5-8.1) |  | 13237 (20553-4722) | 17.7 (19.0-16.6) |  | 41845 (61709-24324) | 19.4 (21.0-17.6) |
|  | Early | 194705 (257659-115919) | 10.3 (13.1-6.6) |  | 146892 (163393-138338) | 12.1 (14.4-10.5) |  | 4842 (6059-2878) | 14.2 (15.5-12.9) |  | 8995 (15968-5966) | 12.8 (14.4-11.4) |
|  | Late | 110005 (144271-75739) | 12.8 (13.1-12.5) |  | 145611 (148073-143148) | 99.7 (185.0-14.4) |  | 45482 (54742-36221) | 87.0 (88.9-85.0) |  | 5735 (9481-1988) | 19.9 (24.7-15.0) |
| **CD3+** | Cont. | 71132 (113729-41234) | 3.5 (3.8-3.3) |  | 76761 (87655-49188) | 5.3 (6.2-4.6) |  | 13786 (24687-9846) | 4.9 (6.2-3.9) |  | 14409 (22646-9821) | 5.2 (7.8-3.4) |
|  | Pre | 113529 (133739-98700) | 3.4 (3.5-3.3) |  | 87094 (97359-82790) | 5.2 (5.3-5.0) |  | 18772 (29143-11975) | 6.5 (6.9-6.1) |  | 19075 (23995-15701) | 8.4 (9.0-7.4) |
|  | Early | 157904 (218293-96610) | 4.3 (4.8-3.7) |  | 62313 (63174-60881) | 5.6 (6.3-5.1) |  | 7728 (15541-3428) | 4.6 (5.8-3.9) |  | 3222 (7247-867) | 3.7 (3.9-3.5) |
|  | Late | 90447 (95113-85781) | 5.4 (6.0-4.8) |  | 48936 (50614-47257) | 4.1 (4.5-3.8) |  | 20591 (20987-20194) | 16.6 (20.7-12.4) |  | 1220 (1556-884) | 3.9 (4.1-3.8) |
| **CD3+**  **CD4+** | Cont. | 41501 (65265-23434) | 9.6 (9.9-9.2) |  | 46243 (56105-26289) | 8.9 (9.6-8.0) |  | 8292 (15939-5387) | 15.6 (19.7-12.4) |  | 6905 (11367-4368) | 14.5 (22.3-9.1) |
|  | Pre | 70555 (81115-61623) | 9.3 (9.6-9.0) |  | 52830 (61307-49011) | 8.5 (8.9-8.1) |  | 11766 (18011-9475) | 19.2 (20.9-17.8) |  | 10242 (12572-8847) | 24.7 (26.2-22.7) |
|  | Early | 89934 (130631-55215) | 11.1 (16.7-7.4) |  | 41762 (42754-41100) | 11.4 (16.0-9.6) |  | 3651 (8051-1397) | 16.0 (17.4-14.8) |  | 1793 (3953-417) | 12.5 (13.2-11.7) |
|  | Late | 53181 (58683-47679) | 18.9 (21.0-16.7) |  | 30777 (33842-27711) | 17.5 (18.7-16.2) |  | 10934 (10985-10882) | 110.0 (113.0-107.0) |  | 674 (824-523) | 19.2 (19.9-18.4) |
| **CD3+ CD8+** | Cont. | 28176 (46310-16987) | 7.1 (11.4-5.7) |  | 27301 (29855-20571) | 6.6 (7.8-5.7) |  | 2906 (4161-2040) | 10.3 (14.3-7.6) |  | 7047 (10727-5109) | 11.5 (24.0-2.3) |
|  | Pre | 41051 (50627-35357) | 6.2 (6.9-5.7) |  | 30899 (33406-27786) | 8.2 (8.6-7.9) |  | 3767 (5901-2334) | 14.4 (15.3-13.7) |  | 8320 (10669-6529) | 26.4 (28.4-24.8) |
|  | Early | 62998 (80379-39089) | 12.6 (23.0-7.7) |  | 18899 (19802-18165) | 9.9 (20.2-6.2) |  | 2306 (4212-1217) | 11.3 (14.6-8.2) |  | 1282 (3182-406) | 6.6 (8.1-5.9) |
|  | Late | 35262 (36350-34174) | 23.9 (24.8-23.0) |  | 17249 (18404-16094) | 18.0 (19.4-16.5) |  | 5084 (5192-4975) | 103.5 (107.0-100.0) |  | 501 (654-348) | 19.2 (19.9-18.4) |
| **pNK46+** | Cont. | 1678 (2866-1012) | 12.2 (13.3-11.2) |  | 8871 (10444-7718) | 10.5 (12.1-9.5) |  | 4895 (6873-3264) | 16.3 (20.5-13.9) |  | 2394 (3145-1343) | 16.4 (26.7-8.9) |
|  | Pre | 1495 (2022-1224) | 11.7 (12.1-11.2) |  | 10800 (14698-9020) | 9.9 (10.6-9.4) |  | 5417 (7937-3304) | 19.6 (21.2-17.8) |  | 2854 (3599-1971) | 27.7 (29.2-25.0) |
|  | Early | 7014 (10296-4551) | 13.9 (20.0-9.9) |  | 7301 (7590-6878) | 16.6 (23.2-13.0) |  | 22463 (27726-14178) | 21.0 (24.8-18.4) |  | 1132 (1885-565) | 16.3 (17.2-15.6) |
|  | Late | 4264 (5069-3458) | 17.1 (20.0-14.1) |  | 4973 (5085-4860) | 65.6 (111.0-20.2) |  | 16497 (17961-15033) | 157.0 (164.0-150.0) |  | 677 (1218-136) | 30.8 (33.6-27.9) |
| **Ly6G+** | Cont. | 279 (464-127) | 181.8 (255.0-127.0) |  | 1873 (2702-1354) | 10.8 (12.6-8.6) |  | 126 (184-109) | 66.7 (96.9-47.0) |  | 6873 (9700-4351) | 22.6 (31.1-11.7) |
|  | Pre | 227 (520-112) | 115.1 (154.0-81.1) |  | 1404 (1895-985) | 11.4 (12.4-10.4) |  | 214 (294-91) | 88.6 (111.0-63.0) |  | 6691 (8957-3467) | 41.1 (51.6-28.4) |
|  | Early | 3397 (5299-2361) | 77.7 (108.0-56.7) |  | 6475 (7827-4825) | 18.3 (33.0-10.7) |  | 2822 (6345-726) | 26.4 (44.8-19.0) |  | 15400 (24638-11334) | 14.4 (18.6-10.7) |
|  | Late | 3439 (4305-2572) | 141.5 (158.0-125.0) |  | 3837 (5010-2663) | 149.0 (187.0-111.0) |  | 34507 (42521-26493) | 625.0 (669.0-581.0) |  | 9205 (14469-3941) | 100.8 (102.0-99.5) |
| **CD11b+** | Cont. | 2658 (7311-790) | 31.5 (44.4-21.3) |  | 11682 (14409-9369) | 26.4 (42.1-17.5) |  | 7432 (10751-5285) | 21.2 (34.5-9.5) |  | 10255 (14431-6843) | 40.2 (71.2-24.0) |
|  | Pre | 2187 (3471-1570) | 31.6 (44.8-17.9) |  | 12469 (16818-8903) | 23.9 (31.2-16.3) |  | 9746 (13615-5772) | 36.8 (42.7-30.7) |  | 10949 (16617-7411) | 64.6 (76.0-38.1) |
|  | Early | 19147 (33536-5337) | 98.3 (133.0-60.2) |  | 16906 (18935-13122) | 48.3 (65.9-36.2) |  | 64459 (109693-23270) | 53.9 (81.4-6.1) |  | 5618 (8806-3279) | 36.0 (53.5-23.6) |
|  | Late | 6220 (9849-2591) | 162.2 (230.0-94.3) |  | 13968 (16506-11429) | 95.9 (106.0-85.7) |  | 50539 (50916-50161) | 633.5 (635.0-632.0) |  | 3813 (6771-854) | 261.0 (316.0-206.0) |
| **CD11b+ Ly6C-** | Cont. | 2209 (6080-646) | 23.9 (81.2-11.3) |  | 6408 (7773-4844) | 11.3 (12.0-10.6) |  | 2926 (4018-1836) | 17.6 (22.5-13.1) |  | 2823 (4076-1843) | 32.4 (49.3-18.1) |
|  | Pre | 1611 (2084-1361) | 13.1 (13.8-12.5) |  | 6595 (8045-5479) | 11.4 (11.7-11.0) |  | 4086 (5877-2621) | 20.8 (22.2-19.4) |  | 3200 (3734-2802) | 44.6 (48.4-40.2) |
|  | Early | 8337 (11493-3461) | 16.4 (17.4-14.7) |  | 5948 (6572-5379) | 15.0 (16.2-13.9) |  | 20759 (27437-11471) | 23.2 (28.0-20.8) |  | 2732 (5674-1087) | 15.8 (18.4-12.5) |
|  | Late | 3621 (5814-1428) | 36.1 (45.7-26.5) |  | 7512 (8162-6861) | 33.5 (39.0-28.0) |  | 13256 (13824-12688) | 256.5 (267.0-246.0) |  | 2552 (4862-241) | 69.1 (72.2-65.9) |
| **CD11b+ Ly6C+** | Cont. | 417 (1145-131) | 23.9 (79.7-11.6) |  | 5193 (7252-3909) | 12.1 (12.9-11.4) |  | 4462 (6666-3194) | 19.3 (24.7-14.3) |  | 7380 (10266-4432) | 34.8 (51.3-19.7) |
|  | Pre | 551 (1344-193) | 13.3 (14.4-12.5) |  | 5803 (8671-3374) | 12.3 (12.7-11.8) |  | 5591 (7643-3114) | 22.4 (24.1-20.7) |  | 7679 (12794-4557) | 47.8 (51.8-43.5) |
|  | Early | 10379 (21276-1787) | 17.0 (18.2-16.3) |  | 10732 (13312-6874) | 16.8 (18.0-16.1) |  | 42980 (84230-11362) | 24.6 (29.3-20.4) |  | 2854 (4107-2039) | 18.9 (20.7-18.1) |
|  | Late | 2437 (3747-1126) | 33.3 (40.5-26.0) |  | 6122 (7954-4290) | 26.4 (28.2-24.5) |  | 36343 (36346-36339) | 159.0 (166.0-152.0) |  | 1234 (1863-604) | 38.4 (44.8-32.0) |
| **Lin- CD45+ Ly6G- CD11b+ MHCII+** | Cont. | 197 (399-93) | 20.7 (35.9-15.1) |  | 247 (504-157) | 16.7 (19.2-14.2) |  | 318 (455-176) | 27.8 (37.1-19.9) |  | 129 (172-92) | 40.2 (69.7-19.1) |
|  | Pre | 114 (148-68) | 18.4 (18.8-17.6) |  | 153 (195-106) | 15.4 (15.8-14.3) |  | 336 (432-256) | 36.9 (40.0-34.3) |  | 84 (129-56) | 66.6 (69.3-59.4) |
|  | Early | 2300 (3592-371) | 24.7 (28.9-21.0) |  | 896 (1214-572) | 20.5 (23.0-17.1) |  | 2615 (4069-1481) | 34.5 (41.5-27.0) |  | 100 (191-42) | 25.5 (27.6-23.8) |
|  | Late | 1042 (1801-282) | 31.0 (35.5-26.4) |  | 1390 (1576-1204) | 31.6 (36.3-26.9) |  | 2120 (2387-1852) | 366.0 (412.0-320.0) |  | 209 (407-11) | 85.0 (110.0-60.0) |

CD19+, B-cells, CD3+, T cells; CD3+CD4+, CD4+ T cells; CD3+CD8+, CD8+ T cells; pNK46+, natural killer cells; Ly6G+, polymorphonuclear neutrophils; CD11b+, monocytes/macrophages; CD11b+ Ly6C-, immature monocyte/macrophages; CD11b+Ly6C+, activated monocyte/macrophages; Lin- CD45+Ly6G- CD11b+ MHCII+, dendritic cells. Total animals in each experimental group include: control, *n* = 6; pre-clinical, *n* = 4; early, *n* = 4; and late, *n* = 2.
