# Supplementary material for: Fluorescent Crimean-Congo hemorrhagic fever virus illuminates tissue tropism patterns and identifies early mononuclear phagocytic cell targets in Ifnar-/- mice
Source: PLoS Pathog. 2019 Dec 2;15(12):e1008183. doi: 10.1371/journal.ppat.1008183 (PMC6984736; doi:10.1371/journal.ppat.1008183)
Supplement: S3 Table — All antibodies from Biolegend (San Diego, CA, USA). (DOCX) [file ppat.1008183.s007.docx]

**S3 Table.** Antibodies used in flow cytometric analyses. All antibodies from Biolegend (San Diego, CA, USA).

|  | **Target** | **Clone** | **Fluorophore** | **Dilution** | **Catalogue number** |
| --- | --- | --- | --- | --- | --- |
| **Lymphocyte panel** | | | | | |
|  | CD3 | 17A2 | APC | 1 to 200 | 100236 |
|  | CD4 | RM4-4 | PCPCy5.5 | 1 to 200 | 116012 |
|  | CD8a | 53-6.7 | BV510 | 1 to 100 | 100752 |
|  | CD335 (NKp46) | 29A1.4 | PECy7 | 1 to 50 | 137618 |
|  | CD19 | 6D5 | PE | 1 to 100 | 115508 |
| **APC panel** | | | | | |
|  | CD11b | M1/70 | BV421 | 1 to 100 | 101251 |
|  | CD45 | 30-F11 | BV785 | 1 to 50 | 103149 |
|  | CD11c | N418 | PECy7 | 1 to 100 | 117318 |
|  | I-Ab | AF6-120.1 | PE | 1 to 200 | 116408 |
|  | F4/80 | BM8 | APC | 1 to 200 | 123116 |
|  | Ly6C | HK1.4 | BV510 | 5 µL/test | 128033 |
|  | Ly6G | 1A8 | PCP Cy5.5 | 1 to 100 | 127616 |
|  | CD3 | 17A2 | APC/Cy7 | 1 to 100 | 100222 |
|  | CD19 | 6D5 | APC/Cy7 | 1 to 100 | 115530 |
